# Variation in metabolic responses to meal challenges differing in glycemic index in healthy women: Is it meaningful?

**DOI:** 10.1186/1743-7075-9-26

**Published:** 2012-03-29

**Authors:** Sridevi Krishnan, John W Newman, Tara A Hembrooke, Nancy L Keim

**Affiliations:** 1 Department of Nutrition, University of California, Davis, CA, USA; 2 USDA, Agricultural Research Service, Western Human Nutrition Research Center, Obesity and Metabolism Research Unit, Davis, CA, USA; 3 Platinum Performance Inc, Buellton, CA, USA; 4 USDA, ARS, Western Human Nutrition Research Center, 430 W. Health Sciences Drive, Davis, CA 95616, USA

**Keywords:** Glycemic index, Phenotyping, Meal challenge tests, Range scaling, Principal component analysis

## Abstract

**Background:**

Established clinical tests are commonly used in disease diagnosis, but tools that enhance identification of metabolic dysfunctions are needed. This study was conducted to identify typical and atypical metabolite temporal patterns in response to paired meal challenge tests.

**Design:**

Metabolic responses to high and low glycemic index (GI) meals were tested in 24 healthy pre-menopausal women, aged 20-50 y, with BMI of 25-30 kg/m^2 ^using a cross-over design. On test days, blood glucose, insulin, leptin and non-esterified fatty acids were measured after an overnight fasting, and for 8 h following test meal consumption. The data were range scaled, and multivariate statistics were used to assess the presence of distinct response groups to the meal challenge tests.

**Results:**

As expected, participants showed higher circulating glucose and insulin in response to the high GI compared to the low GI meal challenge. However, using range-scaling and Principal Component Analysis, three distinct groups were identified based on differential responses to the paired challenges. Members of the most populated group (n = 18) displayed little deviation from the expected response to the two meal challenges. Two minor groups (n = 3/group) with distinct responses were observed, one suggestive of sub-clinical insulin resistance, and the other suggestive of hyperleptinemia.

**Conclusions:**

The differential responses of glucose, insulin and leptin to low and high glycemic test meals revealed three response groups. Dietary intervention studies traditionally evaluate group responses, and aim to identify the overall effect in the population studied. In contrast, our study analyzed the variance in the meal challenge responses, using an integrated physiological approach, rather than a reductionist approach. This phenotyping approach may be useful for detecting subclinical metabolic dysfunctions, and it could contribute to improved personalized nutrition management. This study is registered in ClinicalTrials.gov, record #200210295

## Introduction

Meal challenge tests are common tools used to identify metabolite response patterns in human studies. Populations can also be segregated into specific metabolic phenotypes or *metabotypes *based on their response to a fixed dietary exposure [1]. Recent evidence suggests that the postprandial lipid profile of individuals can be a means to achieve metabolic phenotyping, using postprandial time course data, and statistical tools [2].

The postprandial glycemic response to ingesting carbohydrate-containing foods can be highly variable between individuals, especially in those with impaired glucose tolerance [3]. The postprandial glycemic surge is tempered by an individual's ability to secrete adequate insulin, and clearance of glucose depends on insulin sensitivity of tissues. Downstream, the homeostatic regulation of fuel utilization and storage is also subject to dynamic control through the interaction of different hormonal mediators. For instance, leptin, an endocrine hormone primarily secreted from the adipose tissue, plays an integral part in the hypothalamic regulation of energy homeostasis [4], and circulates in direct proportion to body fat mass [5]. However, variations in leptin concentration in individuals with comparable adiposity suggest that other factors regulate leptin production and release [6].

Numerous studies have indicated that insulin can act as a regulator of both adipose leptin secretion and circulating leptin concentrations [7-10]. Insulin-dependent activation of the insulin receptor in adipocytes increases leptin mRNA expression within a few hours [11]. Also, leptin has been shown to inhibit the pancreatic beta cell secretion of insulin [12], thereby creating a bidirectional circuit of hormone interplay termed the adipo-insular axis. Leptin suppression of insulin is speculated to be involved with long-term regulation of basal insulin secretion [13].

Recent evidence indicates a role for leptin resistance and hyperleptinemia in the metabolic dysfunction that leads to diabetes [14]. Leptin resistance in the hypothalamic center can disrupt body weight regulation [15]. Leptin resistance in pancreatic beta cells can disrupt leptin suppression of insulin via the adipo-insular axis and promote hyperinsulinemia [16].

Here we have used low and high glycemic index meal challenges in an endeavor to identify response patterns that can provide insight into early metabolic disruption. By investigating glucose, insulin and leptin responses to these meals, we observed the expected differential glycemic responses [17]. Further, we applied novel data treatments and achieved a clear stratification of individuals based on their response patterns to low and high GI meals.

## Subjects and methods

### Subjects

Twenty-four women between the ages of 20 to 50 years of age participated after giving informed consent and passing initial health screening consisting of medical history, clinical blood chemistries, and blood pressure measurements. All volunteers were overweight with body mass index (BMI) in the range of 25.0 to 29.9 kg/m^2^. Volunteers were excluded if they had indications of cardiovascular or metabolic disorders, were pregnant or had been pregnant within 18 months prior to the study, were taking medications or herbal supplements to induce weight loss or changes in appetite, or were smokers. Volunteers agreed to remain weight stable during the period of the study and refrain from restrictive dieting or changes in their habitual physical activity level.

### Study design

A cross-over design was used to measure the glucose, insulin, leptin and NEFA responses to meals with high glycemic index (HGI) and low glycemic index (LGI). Healthy, overweight female volunteers were randomly assigned to a test sequence: either HGI meal followed by LGI meal or LGI meal followed by HGI meal. There was a washout period of at least 1 month between test sessions. Before each test session subjects consumed a run-in diet (with glycemic index matching the test meal assignment) for 3 consecutive days, and the test day was scheduled on the 4^th ^day. The macronutrient composition of run-in diets and test meals are described in Table 1. The diet and meals were designed to comply with the Institute of Medicine's acceptable macronutrient distribution range (AMDR) [18]. The study was approved by the Institutional Review Board at the University of California, Davis.

**Table 1:** Composition of Run-In Diets^1 ^and Test Meals^2^

	High GI Diet		Low GI Diet	
	
	Run-In Diet	Test Meal	Run-In Diet	Test Meal
Energy, kcal	2091	833	2106	835

% carbohydrate	56.4	54.2	56.5	54.6

% protein	13.9	15.0	13.9	14.7

% fat	29.7	30.8	29.5	30.7

Fiber, g	9.9	2.4	46.6	35.5

Glycemic Index^3^	76.6	76.7	42.5	36.5

Glycemic Load	225.7	86.3	126.4	42.1

^1^Run-in diet values reported as average of intake per day, based on an energy intake of 2100 kcal/d. The energy content of the run-in diet was adjusted on an individual basis to meet the individual's daily energy requirement for weight maintenance, calculated using the Harris-Benedict equation [27] and adjusted with an activity factor of 1.4

^2^Test meal values represent an energy intake prescription of 2100 kcal/d. The energy content of the test meal was adjusted on an individual basis and provided 40% of the individual's daily energy requirement for weight maintenance. The amounts of foods served in the test meal were adjusted proportionately to maintain the same macronutrient ratios at all energy levels

^3^Glycemic index values [28] are based on the glucose standard and represent an average weighted by available carbohydrate in each food item constituting the test meal or run-in diet

### Test day protocols

Following each 3-d period of consuming the run-in diets, participants reported to the human studies laboratory at 0700 after an overnight, 12-h fast. Their height and weight were measured using a stadiometer and a weight scale. Body composition for fat and fat free mass was measured using a DEXA scan. Then an intravenous catheter was inserted to sample blood at 10 specific time points: 0 min (fasted state); time of blood draw was 0800, and 30, 60, 90, 120, 150, 210, 270, 360, and 480 min following the test meal that was consumed between 0900 and 0915. Indwelling catheters were flushed periodically with normal saline.

### Blood collection and analyses

All blood samples were collected in vacutainers containing sodium fluoride/potassium oxalate for glucose determinations, K_3 _EDTA for leptin determinations, or no additives for insulin and non-esterified fatty acid (NEFA) determinations. To obtain serum, blood was allowed to stand at room temperature for 10 min before centrifuging, whereas blood samples for plasma were immediately chilled on ice. All samples were centrifuged in a refrigerated Centra CL3R centrifuge (International Equipment Co. Chattanooga, TN) for 10 min at 1300 g. Plasma and sera were stored at -70°C prior to analyses. Glucose and NEFA were determined via enzymatic assays (Roche Diagnostics Corp., Indianapolis, IN) using a Hitachi 902 Automatic Analyzer (Boehringer Manheim Corp., Indianapolis, IN). Serum insulin was determined via a solid-phase, two-site, chemiluminescent enzyme-labeled immunometric assay using an Immulite analyzer (Diagnostic Products Corp., Los Angeles, CA). Plasma leptin was determined using a radioimmunoassay (Millipore/Linco, Billerica, MA).

### Data transformation and statistical analyses

To reduce the impact of inter-individual variability in clinical parameters, the matrix of measured raw data was range-scaled prior to analysis by principal component analysis (PCA). Range-scaling assigns scores between 0 and 1 to individual data points based on the inherent scale set by the range of the dataset. Two range-scaling approaches were evaluated for the transformation of glucose, insulin, and leptin data. First, data was centered to the subjects mean value across both test days and all time points and scaled to the maximum range of each subjects measured data [19] as shown in Eq. 1:

(1) $$\frac{\left(x_{ij}{-}^{T}{\bar{x}}_{ij}\right)}{{(}^{T}x_{ij\max}{-}^{T}x_{ij\min})}$$

where, *x**_ij _***is the *i*th element (i.e. subject) in the *j*th column (i.e. metabolites over time) from the data array, $\bar{x}$ is the mean, *x**_ij max _***and *x**_ij min _***are the maximum and minimum in the metabolite temporal data array. The second approach "adjusted" the range for each subjects' data by first reducing each individuals test day responses by that days nadir response, followed by scaling each individual's response to both the meal challenges to the HGI nadir adjusted response.

(2) $$\frac{{(}^{t}x_{ij}{-}^{t}x_{ij\min})}{{(}^{HGI}x_{ij\max}{-}^{HGI}x_{ij\min})}$$

This "adjusted range-scaling" presumes that the daily minimum reports on an individuals' basal status, and that the HGI meal represents a maximum challenge response, and together these represent the best measure of an individuals' ability to respond to a glycemic challenge.

A PCA was performed on the resulting data sets of leptin, insulin and glucose to assess variance in metabolic response. A PCA transforms a data matrix (*e.g*. metabolite array by subject) into a set of uncorrelated variables, or principal components, that are ranked by their ability to describe the maximum variance in the data, and being uncorrelated (*i.e*. orthogonal or perpendicular) to the previous principal component, with each subject receiving a score based on their number of metabolite input data in each principal component [20]. The degree of influence each variable has within a given principal component is reported by the loadings for that variable. Thus, PCA analyzes variance in the dataset, and scores each participant based on this variance. In this case, PCA plots depict the spread of data that has been scaled to enhance the differential response to HGI and LGI in insulin, leptin and glucose. PCA was performed using Bristol Chemometric PCA Add-in http://www.chm.bris.ac.uk/org/chemometrics/ for Microsoft Excel. ANOVAs with Tukeys and Bonferroni's multiple comparison tests were used on raw, range-scaled and area under the curve values (computed using the trapezoid rule) for insulin, leptin and glucose to identify differences.

## Results

### Descriptive anthropometry

Height, weight and body composition did not differ between the two test days (Table 2). All women were overweight with BMI values between 25.0 and 29.9 kg/m^2^, with one exception, a woman with BMI = 24.3 kg/m^2^.

**Table 2:** Characteristics of study population (n = 24) measured on test days^1^

	Weight (kg)	BMI (kg/m^2^)	Age (y)	FFM (kg)	FM (kg)
**HGI**	76.4 ± 6.0	27.2 ± 1.3	30.2 ± 8.4	46.8 ± 4.7	29.7 ± 2.6

**LGI**	76.1 ± 5.8	27.1 ± 1.1	30.4 ± 8.4	46.8 ± 4.4	29.2 ± 2.9

**Range**	66.0 to 89.7	24.3 to 29.0	19.8 to 46.4	38.1 to 58.1	22.6 to 34.6

^1^All results are reported as means ± SD; BMI = Body mass index, FFM = Fat free mass, FM = Fat mass. HGI = High glycemic index and LGI = Low glycemic index

### Blood chemistry

**Figure 1:**
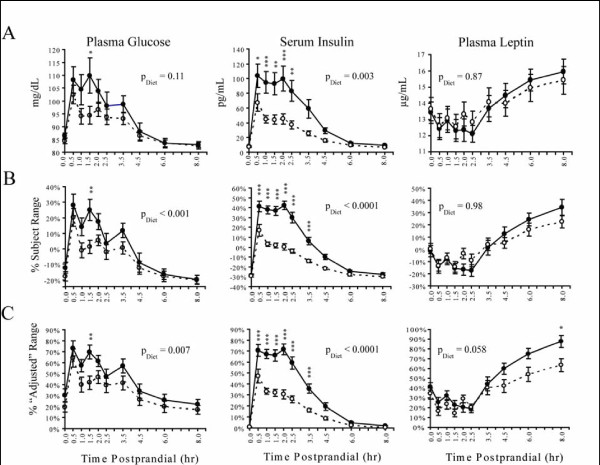
**Concentrations of glucose, insulin, and leptin measured at fasting and postprandial time points after a high GI (●) and low GI (○) challenge**. Performing a diet × time two-way ANOVA of the raw data (Panel A) showed significant diet-dependent differences in insulin (p = 0.003) but not glucose or leptin responses. Range scaling the data (Panel B) significantly reduced the variance at all-time points (p < 0.05, F-test). Adjusting the data by each individual's daily nadir prior to scaling to the subjects experimental HGI range corrected for additional inter-individual variability (Panel C). This "nadir-adjusted" range scaling procedure had little to no effect on assessment of insulin responses, but suggests a diet-dependent difference glucose (p = 0.007) and leptin response (p = 0.058). All results are means ± SEM. Time points showing significant differences in Bonferroni's post-hoc analyses are indicated at p < 0.05 (*), p < 0.01 (**), and p < 0.001 (***).

The average plasma leptin, serum insulin and plasma glucose concentrations for HGI and LGI test days are summarized in Figure 1 panel A. Circulating glucose and insulin concentrations increased by 30 min following both meals, and the postprandial increase in leptin was delayed until > 3 h into the test period.

### Data range-scaling

**Figure 2:**
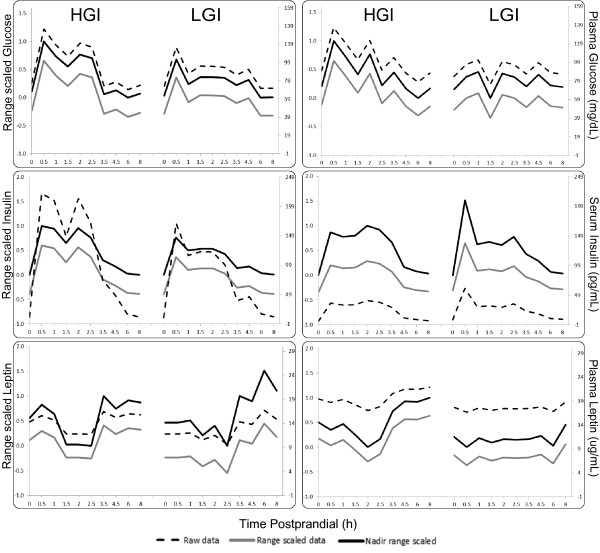
**Data from two randomly chosen subjects illustrating raw, conventional range scaled and nadir-adjust range scaled data for glucose (top), insulin (middle), and leptin (bottom)**. Scale for the raw data is on the right, and scale for the two sets of transformed data is on the left. Note that the trend in data is preserved by the transformations.

The raw, mean centered-range scaled, and nadir-adjusted range-scaled data for insulin, glucose and leptin are compared in Figures 1 and 2. Figure 2 displays a comparison of raw, conventional range-scaled and nadir-corrected range scaled data alongside each other. It shows that the trend and spread of data has not been changed by the transformation techniques employed. Additional files 1 and 2 display individual subject data, and shows this to be true as well. In Figure 1, in the untransformed data, only insulin showed a significant difference (p < 0.01) between the LGI and HGI challenge tests. The range-scaled data reduced the variance at all-time points relative to the raw data (p < 0.05, F-test). With mean centered range-scaling differences in glucose pattern were also detected (p < 0.001) between HGI and LGI challenges. Nadir-adjusted scaling preserved the detected differences in glucose and insulin, while also showing a differential leptin response between diets at a p = 0.06, with significance for the 8 h time point reaching a p < 0.05.

### PCA-Glucose-insulin-leptin response

**Figure 3:**
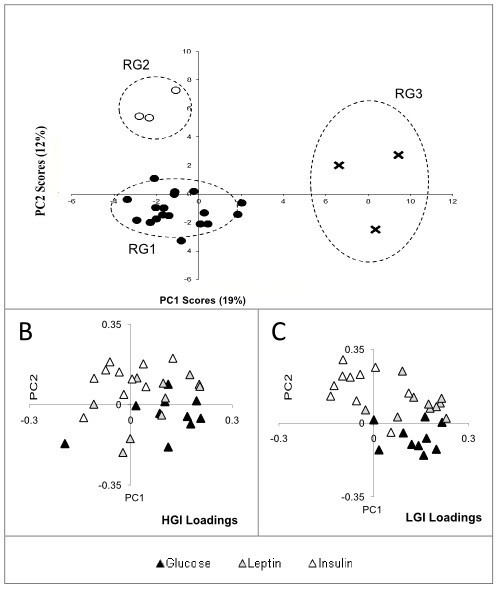
**Panel 'A' is the PCA scores plot of range-scaled HGI and LGI at all time-points for glucose, insulin and leptin responses**. PC1 (x-axis) and PC2 (y-axis) represent 19% and 12% of the total variance in the dataset. While the majority of subjects (n = 18) fall into *Metabolic Group 1- ****RG1 ***(●), the subjects indicated using (○) *Response Group 2- ****RG2 ***and (**x**) *Response Group 3- ****RG3 ***are two distinct groups of subjects that exhibit responses different from RG1. The circles around the RG groups indicate mean ± 2xSD limits. Panel 'B' and 'C' are the loadings plot that correspond to panel 'A' and indicates that the LGI responses are more variant than HGI, and also that while insulin dictates the separation of subjects in one direction (top left quadrant), glucose and leptin appear to be the most variable responses in another direction (top and bottom right quadrants).

A frequency distribution plot of the differential response to low and high GI meals revealed the fact that a sum of 2 Gaussian equations was the best fit, not a single Gaussian distribution (Additional file 3). This indicated the possibility that there were different response groups in the population tested that did not fit the same population. A PCA was done to analyze this variability in response. This PCA using subject nadir-adjusted and range-scaled insulin, leptin and glucose responses between the two meal challenges is shown in Figure 3, and subjects cluster into three groups. The majority of subjects (n = 18) cluster together and are identified here as Response Group 1 (RG1). RG1 displays differences between HGI and LGI meal challenge response profiles consistent with typical glucose and insulin responses [21]. The responses exhibited by the six remaining subjects differed from RG1. (Additional file 4 is a comparison of range-scaled vs raw data PCA displaying the lack of discerning ability of the PCA using the raw data, while the range-scaling showed the groups that were identified)

Compared to RG1, subjects clustering in the upper left PCA quadrant, identified as Response Group 2 (RG2) had lower postprandial leptin, higher insulin and higher glucose relative responses to the two meal challenges (Figure 3). On the other hand, subjects with significantly elevated principal component 1 scores, identified as Response Group 3 (RG3) had high leptin and glucose, with similar insulin responses to RG1.

**Figure 4:**
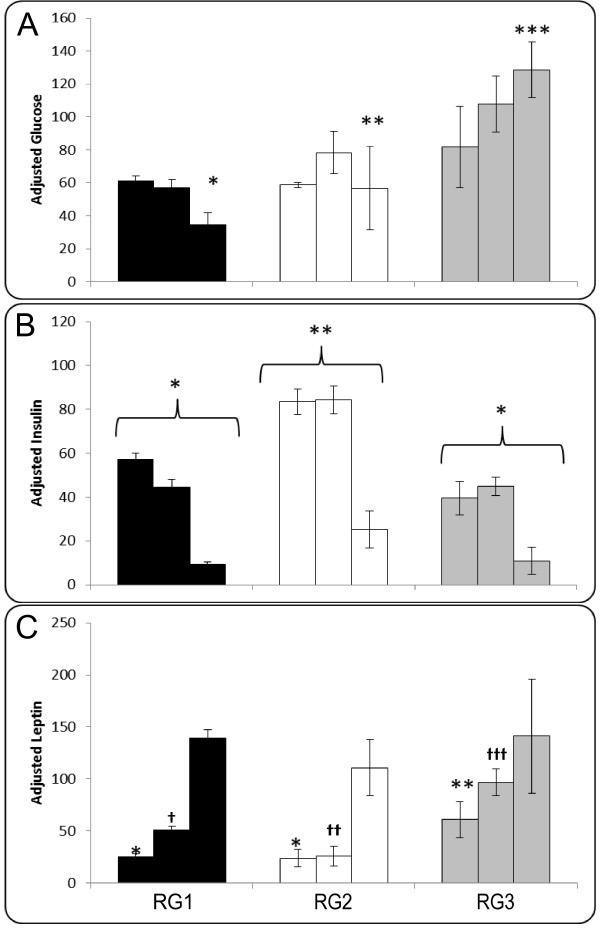
**Panel 'A', 'B' and 'C' are AUC glucose, insulin and leptin composite scores for HGI and LGI range scaled data of Response Groups 1, 2 and 3**. AUC was done using the trapezoid rule for 0-2.5 h, 2.5-4.5 h and 4.5-8 h, and is represented by three bars in sequence for each group. Error bars are ± SEM. A log transformed AUC was used in a 3 × 3 ANOVA with Tukey's post hoc test for RG group (RG1, RG2 or RG3), diet (HGI or LGI) and time (early, mid or late), which revealed a significant main effect of group in leptin, glucose and insulin (p < 0.01). In both glucose and leptin, however, there was a significant interaction between response group and time (p < 0.05), while insulin had no significant 2-way or 3-way interaction. '*', '**', '***' and '†', '††', '†††' indicate significant differences (p ≤ 0.05).

Once these response groups were identified, a closer comparison of their metabolite responses was used to define their profile (Figure 4). A 3-way ANOVA revealed a significant effect of diet for glucose (p < 0.01), insulin (p < 0.01), leptin (p < 0.01) in all groups. And, the group × time interaction was significant (p < 0.01) in glucose and leptin, but not in insulin. Relative to RG1, RG3 is characterized by higher postprandial glucose, higher early and mid-postprandial leptin combined with similar postprandial insulin response. RG2 on the other hand is characterized by lower glucose response than RG3, similar glucose and leptin responses to RG1, but the highest insulin response.

As the final step in characterizing these groups, we examined additional descriptive parameters (Table 3). Body weight, BMI, age and fasting NEFA did not differ between the groups, despite RG3's lower fasting NEFA concentration. Total fat mass was lower in RG2 (p = 0.011) than RG1 and RG3. Table 4 offers a visual summary of characteristics of RG2 and RG3 relative to RG1.

**Table 3:** Metabolic characteristics of RG1, RG2 and RG3

	RG1		RG2		RG3	
	
	Mean ± SEM	Range	Mean ± SEM	Range	Mean ± SEM	Range
Weight (kg)	77.3 ± 1.4	66.0 - 89.7	71.6 ± 0.6	69.3 - 72.9	74.6 ± 2.9	69.2 - 83.7

BMI (kg/m^2^)	27.1 ± 0.3	24.3 - 29.5	26.8 ± 0.5	25.7 - 28.1	27.8 ± 0.5	26.8 - 29.7

Age (y)	29.9 ± 2.0	19.8 - 46.6	27.7 ± 3.2	21.2 - 36.8	35.3 ± 4.7	22.1 - 44.8

*FM (kg)	30.0 ± 0.6^a^	25.7 - 34.6	25.5 ± 1.1^b^	22.6 - 29.0	30.0 ± 1.4^a, b^	27.7 - 34.4

NEFA (mmol/L)	0.60 ± 0.05	0.14 - 0.97	0.51 ± 0.08	0.27 - 0.77	0.35 ± 0.08	0.12 - 0.57

For fat mass, designated by '*', there was a main effect of group (p < 0.05). Means with different superscripts are significantly different using Tukey-Kramer's post-hoc analysis (p = 0.04) as evaluated by a 3 × 2 ANOVA. RG = Response Group; BMI = body mass index; FM = fat mass; NEFA = non-esterified fatty acids

**Table 4:** Summary of RG2 and RG3^1 ^compared to RG1

	RG2	RG3
Glucose Response	**↔**	**⇈**

Insulin Response	**⇈**	**↓**

Leptin Response	**↓**	**↑**

NEFA	**↔**	**↓**

Fat mass	**↓**	**↔**

^1^Arrows indicating up or down are relative to RG1 response, which is considered the expected response. Up arrows (**↑**), such as in insulin in RG2, and glucose and leptin in RG3, indicate that the AUC response was higher relative to RG1, while down arrows (**↓**) indicate the opposite. Horizontal arrows (**↔**) indicate no relative difference. Double arrows indicate significant differences, while single arrows are indicative of the trends observed in data. RG = response group; NEFA = non-esterified fatty acids

## Discussion

Variation in human data can be generated from multiple sources. Identifying deviations between individual responses to dietary challenges and grouping individuals with similar phenotypic responses is one means of "metabotyping." Inter-individual variability in human studies can interfere with the identification of human metabotypes, but appropriate data transformations can facilitate data interpretation. These tools can reduce the volume and complexity of data sets and can quiet variance from both biological and analytical sources. Hence, pre-treatment of data has been widely used to organize data, enabling efficient analysis with improvement in statistical power [19]. In the current study, we transformed data to an individualized response scale to reduce inter-individual variability, which enhanced the ability to identify distinct groups within the measured response profiles. The response profile of RG1 appears consistent with the expected differential response to a low and high glycemic meal challenge. RG2 and RG3 appear to indicate alternate physiological responses within this clinically healthy population. We hypothesize that these distinct "response profiles" are putative metabolic phenotypes that can be identified based on their postprandial response to a single meal challenge.

The range of a dataset is affected by both biologically significant inter-individual variability, and measurement or analytical errors. Range-scaling first centers the data matrix, followed by scaling each data point to the range of the data array. This transforms the dataset into one containing values bound by ± 50% of the mean. Centering reduces offsets of the data set, decreasing the variability introduced by large differences in magnitude, while shifting the scale of the resulting data set by the magnitude of the mean. Once variance is reduced, this cleaner dataset is more proficient at revealing trends that would likely have gone unnoticed in untreated data [22]. When metabolites are analyzed together using a multivariate technique that identifies trends in data based on variance, often the variables that exhibit large changes under similar treatment or challenge conditions tend to dominate the results. In this study-insulin which is highly responsive to the glycemic index of meals could be considered such a metabolite, as opposed to leptin, which might not display such large variations in magnitude. Range-scaling controls for this, by placing each metabolite on a 0-100% scale of its own experimental response range [23]. The data was nadir-corrected for each test day, and scaled to the range of the high glycemic index response in order to better highlight the individual's relative responses to the HGI and LGI challenges. Raw data and mean-centered data failed to show this discrimination. One drawback of range scaling is its sensitivity to outliers in the dataset [19], as is PCA, albeit a conservative multivariate analysis technique. When applying PCA, one must be cautious of outliers in the dataset that define the variance of the distribution instead of the inherent biological variation that is the target. It is also important to note that the PCA was performed on "auto-scaled" data (*i.e*. data transformed to unit variance) prior to the analysis, which allows each variable to have equal weight in the analysis [19]. Performing the analysis on the adjusted-range scaled data without further transformation did reveal RG3 as distinct from RG1 and RG2 (p = 0.11), while RG2 subjects could not be excluded from RG1 using a test for statistical outliers. Mean centering the data improved the RG3 difference (p = 0.001), but increased the variance in the subjects making up the RG2 group. In this dataset, nadir-adjusted range scaling enabled the PCA to identify clustering of individuals with similar postprandial responses better than the raw dataset or the conventional range scaled dataset.

Circulating leptin exhibits a diurnal rhythm and increases about 4-h following a mixed meal [24]. In humans, acute leptin responses to meals-high fat, high carbohydrate, and high GI-have been studied, yielding variable results. High carbohydrate as opposed to high fat meals have been shown to increase leptin secretion [25]. Compared to a low GI meal, a high GI meal has been shown to result in lower postprandial leptin concentrations, but only over a 2-h period [26], a time frame too short to observe insulin-induced leptin secretion.

*In vitro *and animal model studies have suggested that the adipoinsular axis is a long term regulatory circuit that likely plays a role in the metabolic response to meals. The postprandial insulin response is modulated by circulating concentrations of leptin. The theory behind the adipo-insular axis posits that chronic leptin secretion, and its downstream insulin suppression is maintained in normal healthy individuals at an optimal level. Analyzing the insulin, glucose and leptin response to the meal challenges can give insight into the functioning of this axis. The adipo-insular axis can play an important role in establishing fasting and postprandial glycemia, and glucose clearance from circulation.

In this study, the response group designated RG1 (n = 18) is most likely representative of the general population, i.e. normal, healthy individuals with typical GI meal induced responses. On the other hand, RG2 displays the highest insulin AUC profile of the 3 groups associated with a lower leptin response. This relatively high insulin response is irrespective of the meal composition (seen both in HGI and LGI) since the 3 × 3 ANOVA indicated that RG2 had higher HGI insulin response than RG1 and RG3 challenged with either diet and at early- mid- and late-postprandial phases. The relatively elevated insulin response in RG2 could indicate reduced insulin sensitivity as compared to RG1, despite RG2 having a lower fat mass than RG1. Also, while RG1 and RG3 display different responses to the two meal challenges, RG2 displays similar insulin and glucose responses to both the meal challenges. This further is indicative of RG2's errant metabolic fine-tuning to meal composition.

In contrast, relative to RG1, RG3 displays a higher early postprandial leptin AUC, in association with higher postprandial glucose, and a similar insulin AUC response. Since RG3 insulin response is similar to or slightly lower than RG1, but ineffective at maintaining glycemia similar to that of RG1, could indicate either a reduced sensitivity or a higher postprandial suppression of insulin by leptin. It can be speculated that in RG3, the meal challenges elicited a higher leptin response, with stronger suppression of insulin secretion, resulting in lower circulating insulin, and higher glucose concentrations. In addition, this group has a significantly higher fat mass than RG2, although not different from RG1. Postprandial leptin secretion is likely a function of the insulin sensitivity of adipose tissue. Ideally, in response to a HGI meal, the higher insulin secretion will be regulated by leptin, but optimally so as to not disrupt glucose clearance. Thus, impairment in this axis can result in poor glycemic control as exhibited by RG3.

The number of subjects in each of the response groups (n = 3) is small. While individual variation or noise might explain this observation, the three clusters observed in the PCA scores plot of the transformed data could also indicate clustering of similar metabolic response profiles. Further mechanistic studies are necessary to evaluate if the identified groups are distinct phenotypes. Another limitation of the current study is that it is not possible to distinguish the influence of the run in diets on the postprandial meal challenge response that we have characterized. It is likely that the response groups that we have identified are a function of an acclimation to the 3 days of the run in diets, in addition to the meal challenge. Also, the subjects were challenged just once with each of the two meal challenges, and this is a limitation of this study.

Together, these findings suggest that the use of meal challenges and statistical exploratory tools could lead to metabolic phenotyping. This may enable better population sorting to identify health risks associated with specific postprandial phenotypic responses. Scaling, transformations, and non-parametric multivariate analyses have identified subsets from the study population of differing metabolic response profiles. Depending on how similar or dissimilar the individuals' response is to previous literature suggested postprandial metabolic events (which are likely also the majority of the normal, healthy population's responses) a metabolic fingerprint of this variation can be used to classify metabolic status at a finer gradient. Even the current clinically normal responses can be better characterized by using meal challenge responses and statistical tools to identify subgroups of metabolic characteristics that can form the basis of putative phenotypes. Such efforts may ultimately allow greater diagnostic finesse.

## Abbreviations

BMI: Body mass index; LGI: Low glycemic index; HGI: High glycemic index; RG: Response Group; NEFA: Non-esterified fatty acid; PCA: Principal component analysis.

## Competing interests

The authors declare that they have no competing interests.

## Authors' contributions

TAH and NLK designed and conducted the study; SK, JWN, and NLK analyzed data; SK, JWN, and NLK wrote the manuscript; and NLK had primary responsibility for final content. All authors read and approved the final manuscript.

## Note

This study was supported by intramural funds from USDA, ARS, CRIS projects 5306-51000-002-00D and 5306-51530-019-00D.

## Supplementary Material

Additional file 1 **Appendix I A: Raw data for leptin, glucose, and insulin for the 24 subjects that were included in the analysis**. Charts labeled A through R belong to MP1, while S, T, and U are MP2 and V, W, and X are MP3. Leptin, glucose and insulin track each other well as displayed by the temporal response pattern across the three parameters. Click here for file

Additional file 2 **Appendix I B: Range-scaled data for leptin, glucose and insulin for the 24 subjects that were included in the analysis**. Charts labeled A through R belong to MP1, while S, T, and U are MP2 and V, W, and X are MP3. Click here for file

Additional file 3 **Appendix II: Panel A is a repeat of Figure **3**from the paper, and Panel B is the PCA scores and loadings plot of raw, unscaled/untransformed leptin, insulin and glucose data**. In both high and low GI meal challenge responses, the distribution of the subjects in the scores plot appears to be dominated by the fact that the circulating concentration of leptin is very different from glucose and insulin. The scores plot using raw data in B does not afford a clear stratification of subjects, as opposed to panel A which uses nadir adjusted range scaled data where three clusters appear. Click here for file

Additional file 4 **Appendix III: Mean Glucose AUC was lower under LGI conditions (p < 0.001), however the frequency distribution of the differential response to low and high glycemic meals were significantly different than expected (x2 = 6, n = 24, p = 0.014)**. The untransformed frequency distribution was best fit by a sum of 2 Gaussian equations. Using these constraints, 1 hypoglycemic, 16 normoglycemic, and 8 hyperglycemic relative responders were observed. These are highlighted in the inset of the lower figure, being unequally distributed about the mean regression of the HGI vs LGI response. Click here for file
