# Supplementary material for: Variation in metabolic responses to meal challenges differing in glycemic index in healthy women: Is it meaningful?
Source: Nutr Metab (Lond). 2012 Mar 29;9:26. doi: 10.1186/1743-7075-9-26 (PMC3352098; doi:10.1186/1743-7075-9-26)
Supplement: Additional file 1 — Appendix I A: Raw data for leptin, glucose, and insulin for the 24 subjects that were included in the analysis. Charts labeled A through R belong to MP1, while S, T, and U are MP2 and V, W, and X are MP3. Leptin, glucose and insulin track each other well as displayed by the temporal response pattern across the three parameters. [file 1743-7075-9-26-S1.PDF]

Supplemental Figure 1. Raw data for leptin, glucose, and insulin for the 24 subjects that were included in the analysis. Charts labeled A through R belong to MP1, while S, T, and U are MP2 and V, W, and X are MP3. Leptin, glucose and insulin track each other well as displayed by the temporal response pattern across the three parameters.

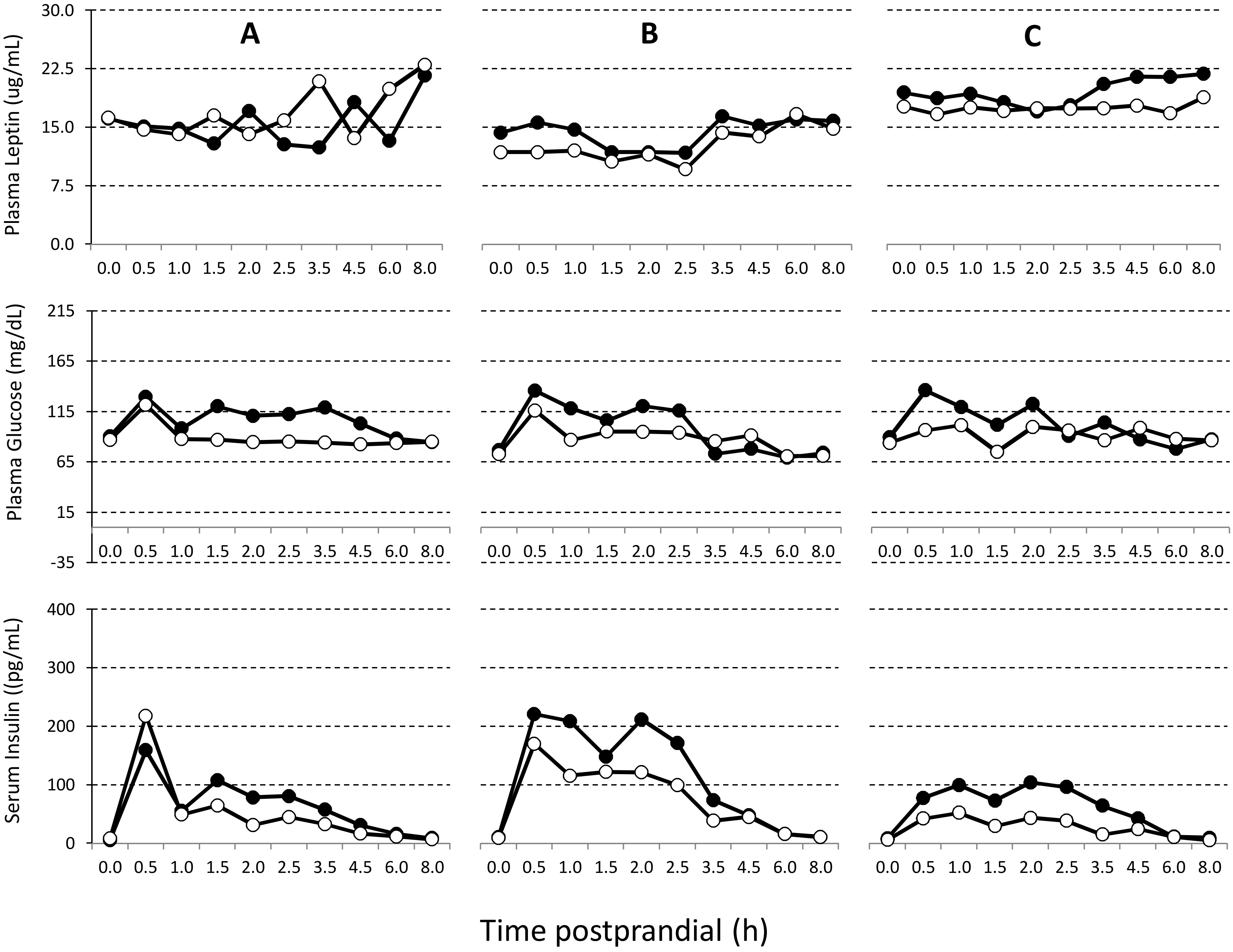
A
B
C
Plasma Leptin (ug/mL)
30.0
22.5
15.0
7.5
0.0
Plasma Glucose (mg/dL)
215
165
115
65
15
-35
Serum Insulin ((pg/mL)
400
300
200
100
0
0.0 0.5 1.0 1.5 2.0 2.5 3.5 4.5 6.0 8.0
Time postprandial (h)

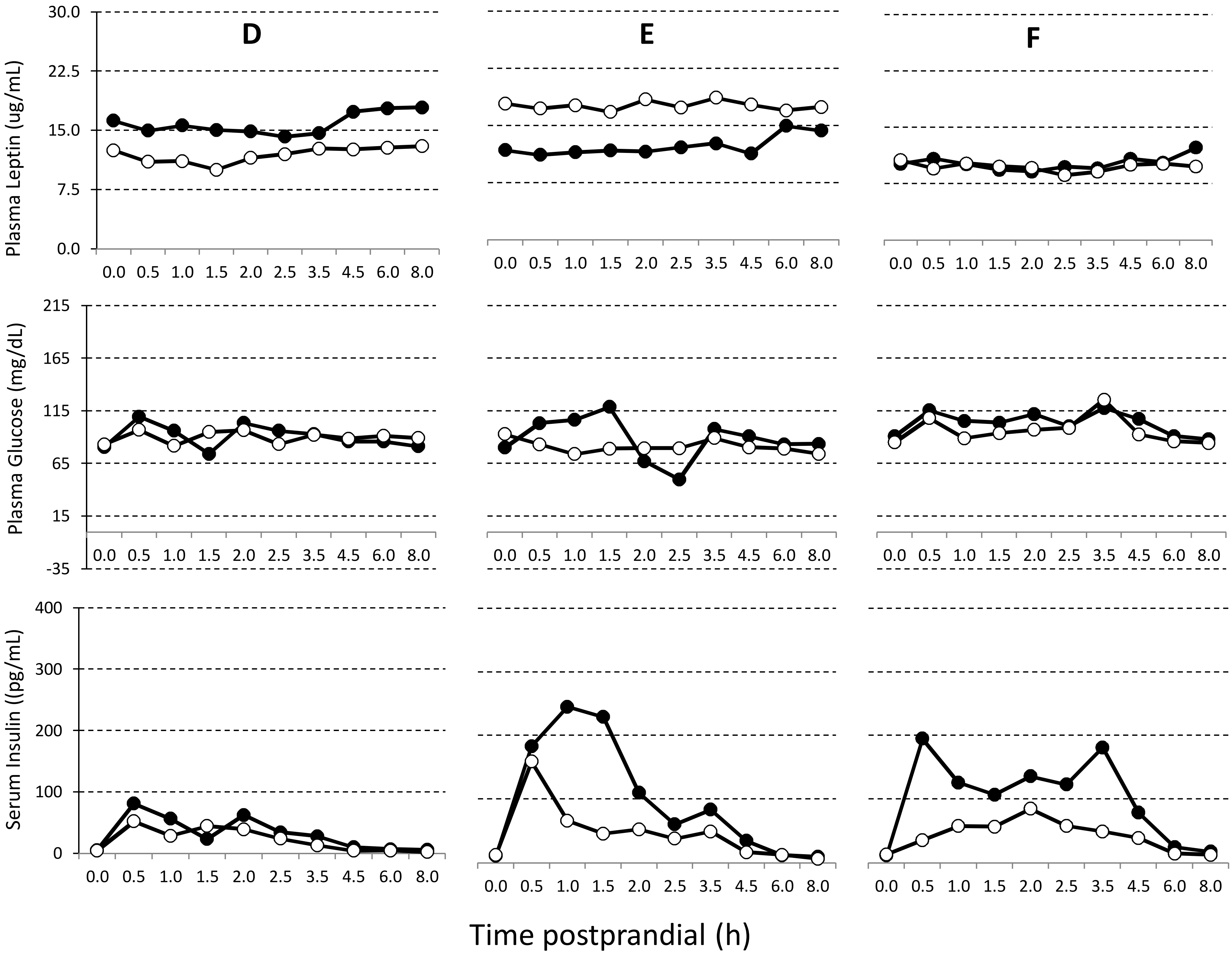
D
E
F
Plasma Leptin (ug/mL)
30.0
22.5
15.0
7.5
0.0
Plasma Glucose (mg/dL)
215
165
115
65
15
-35
Serum Insulin ((pg/mL)
400
300
200
100
0
0.0 0.5 1.0 1.5 2.0 2.5 3.5 4.5 6.0 8.0
Time postprandial (h)

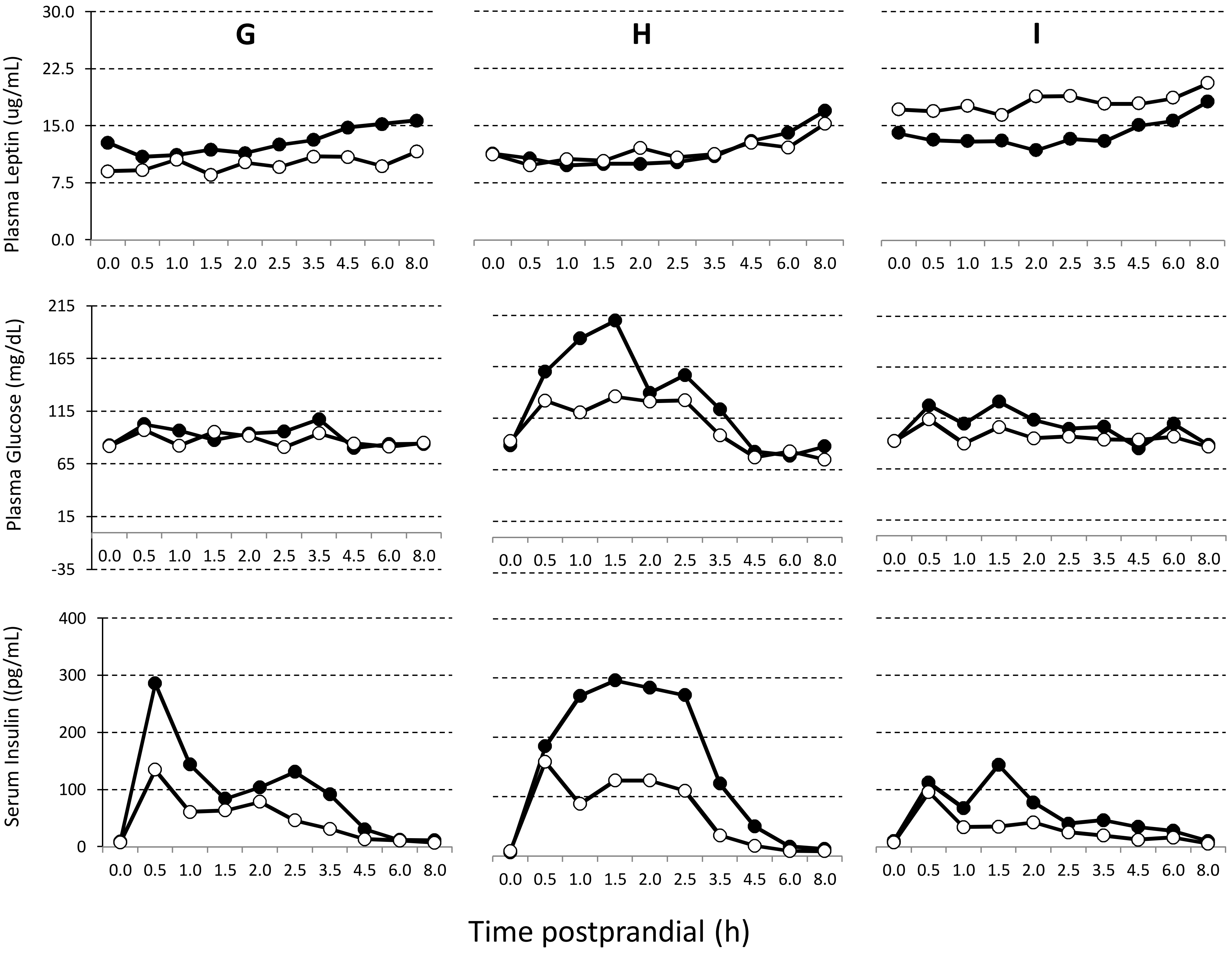

G
H
I
Plasma Leptin (ug/mL)
30.0
22.5
15.0
7.5
0.0
Plasma Glucose (mg/dL)
215
165
115
65
15
-35
Serum Insulin ((pg/mL)
400
300
200
100
0
0.0 0.5 1.0 1.5 2.0 2.5 3.5 4.5 6.0 8.0
Time postprandial (h)

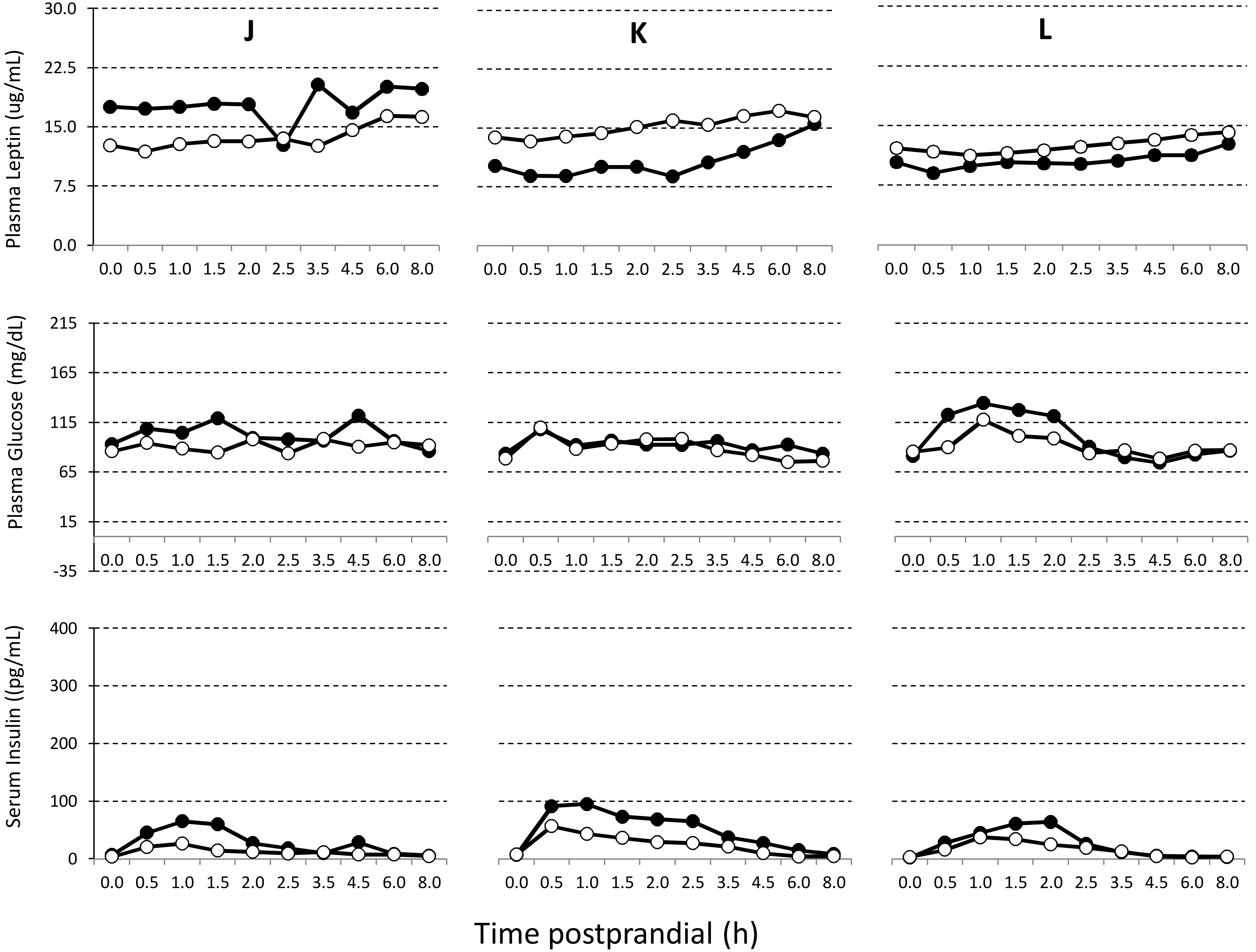

J
K
L
Plasma Leptin (ug/mL)
30.0
22.5
15.0
7.5
0.0
Plasma Glucose (mg/dL)
215
165
115
65
15
-35
Serum Insulin ((pg/mL)
400
300
200
100
0
0.0 0.5 1.0 1.5 2.0 2.5 3.5 4.5 6.0 8.0
Time postprandial (h)

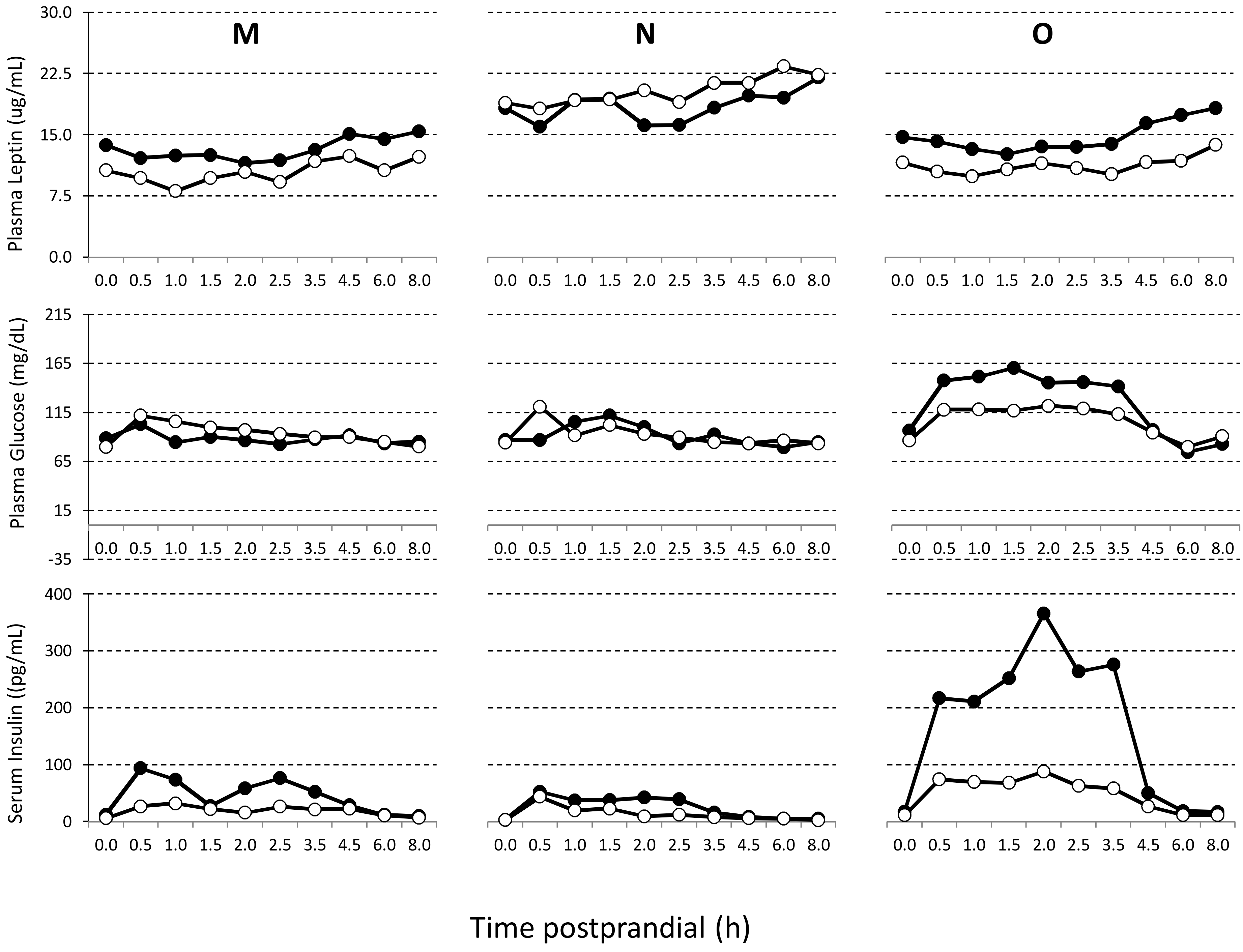

M
N
O
Plasma Leptin (ug/mL)
Plasma Glucose (mg/dL)
Serum Insulin ((pg/mL)
30.0
22.5
15.0
7.5
0.0
215
165
115
65
15
-35
400
300
200
100
0
0.0 0.5 1.0 1.5 2.0 2.5 3.5 4.5 6.0 8.0
Time postprandial (h)

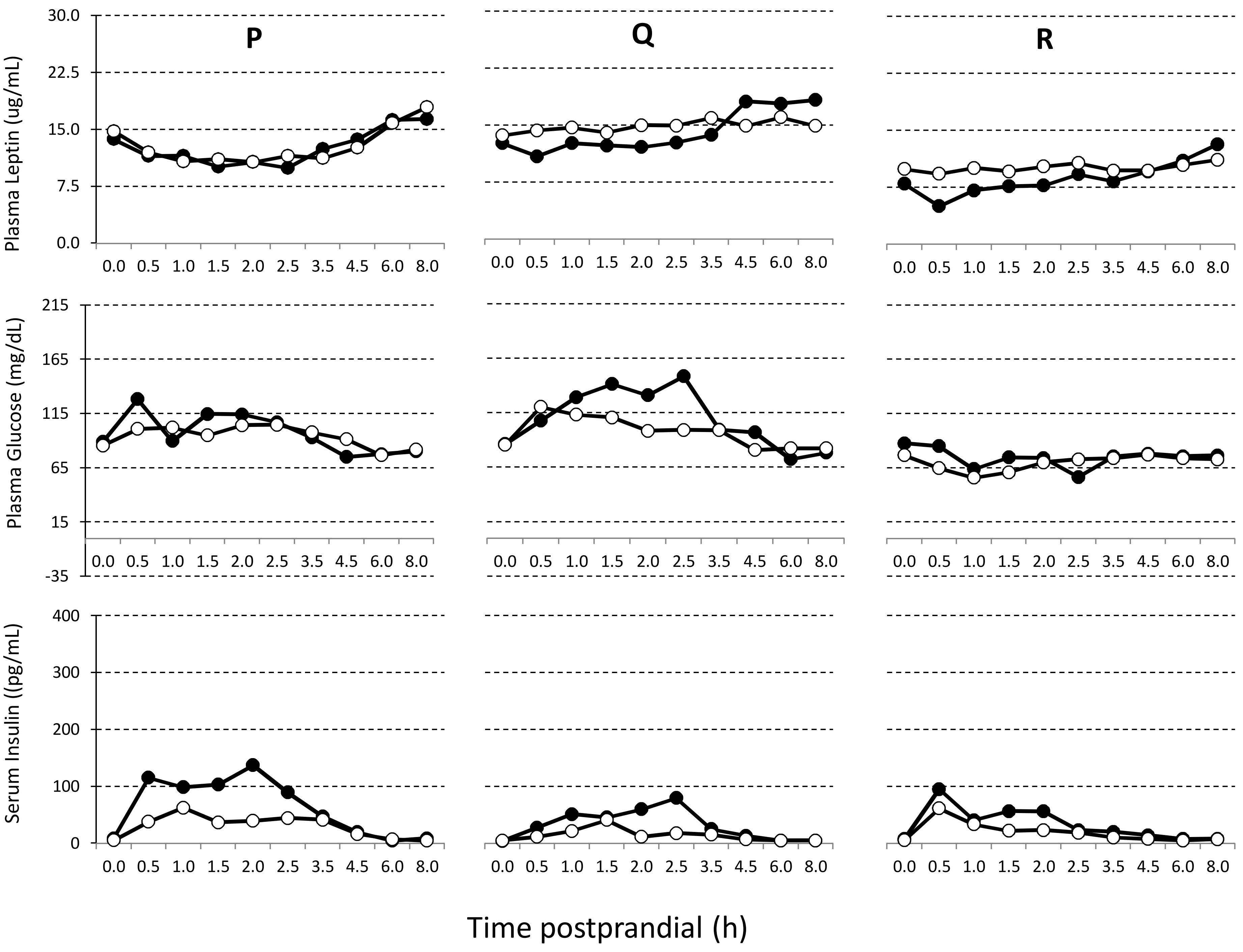
P
Q
R
Plasma Leptin (ug/mL)
Plasma Glucose (mg/dL)
Serum Insulin ((pg/mL)
30.0
22.5
15.0
7.5
0.0
215
165
115
65
15
-35
400
300
200
100
0
0.0 0.5 1.0 1.5 2.0 2.5 3.5 4.5 6.0 8.0
Time postprandial (h)

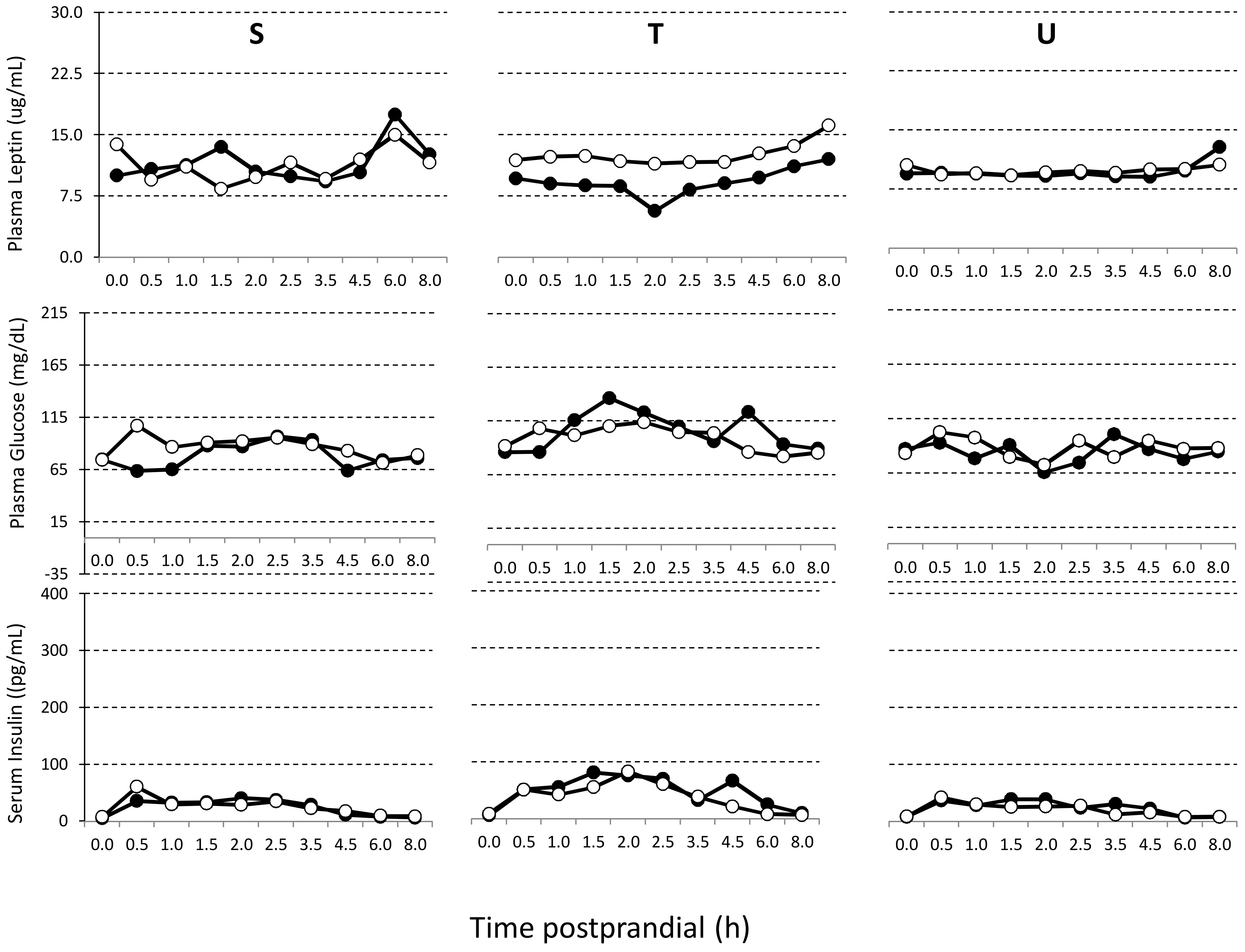
S
T
U
Plasma Leptin (ug/mL)
30.0
22.5
15.0
7.5
0.0
Plasma Glucose (mg/dL)
215
165
115
65
15
-35
Serum Insulin ((pg/mL)
400
300
200
100
0
0.0 0.5 1.0 1.5 2.0 2.5 3.5 4.5 6.0 8.0
Time postprandial (h)

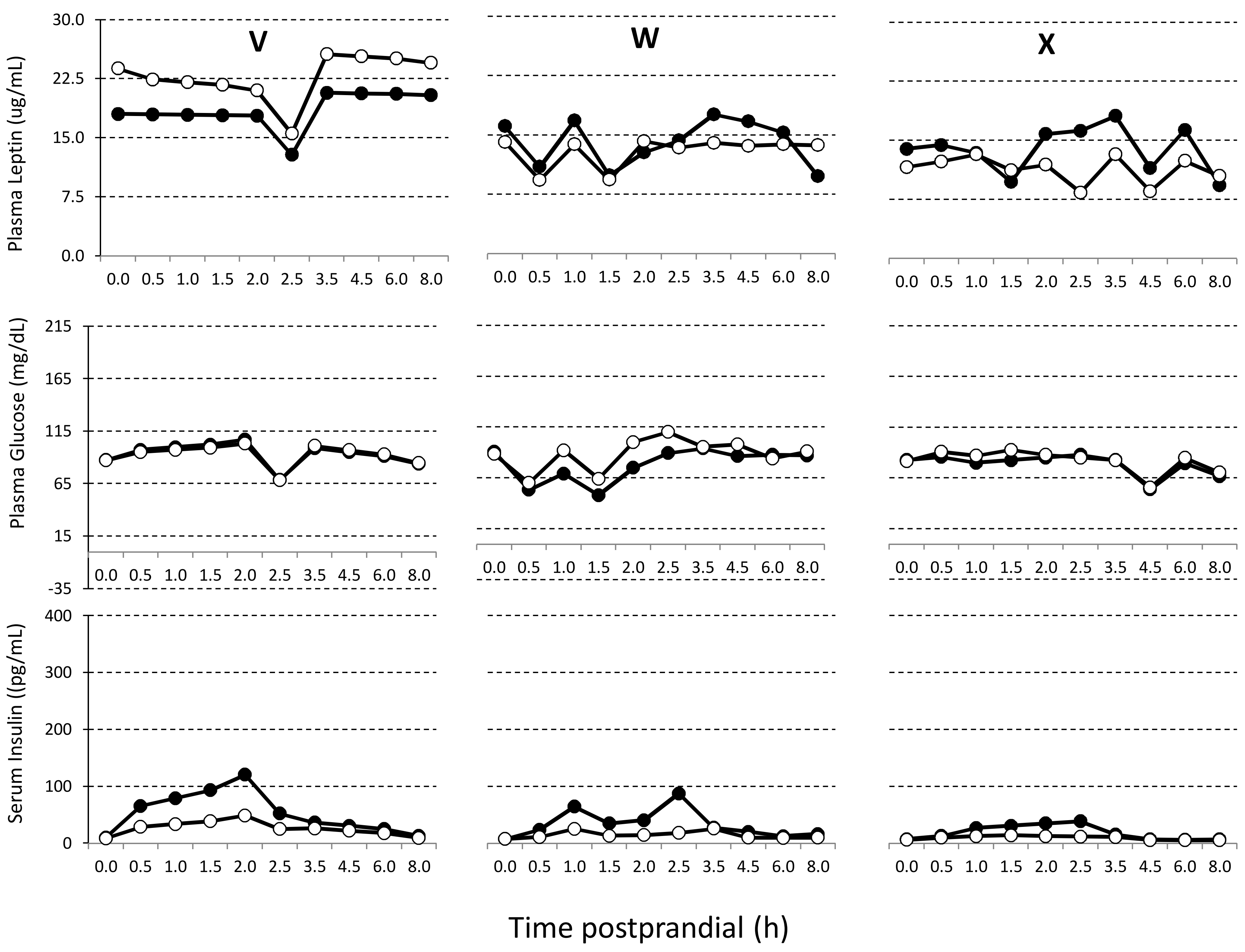

V
W
X
Plasma Leptin (ug/mL)
30.0
22.5
15.0
7.5
0.0
Plasma Glucose (mg/dL)
215
165
115
65
15
-35
Serum Insulin ((pg/mL)
400
300
200
100
0
0.0 0.5 1.0 1.5 2.0 2.5 3.5 4.5 6.0 8.0
Time postprandial (h)
