# Supplementary material for: Variation in metabolic responses to meal challenges differing in glycemic index in healthy women: Is it meaningful?
Source: Nutr Metab (Lond). 2012 Mar 29;9:26. doi: 10.1186/1743-7075-9-26 (PMC3352098; doi:10.1186/1743-7075-9-26)
Supplement: Additional file 2 — Appendix I B: Range-scaled data for leptin, glucose and insulin for the 24 subjects that were included in the analysis. Charts labeled A through R belong to MP1, while S, T, and U are MP2 and V, W, and X are MP3. [file 1743-7075-9-26-S2.PDF]

Supplemental Figure 2. Range-scaled data for leptin, glucose and insulin for the 24 subjects that were included in the analysis. Charts labeled A through R belong to MP1, while S, T, and U are MP2 and V, W, and X are MP3.

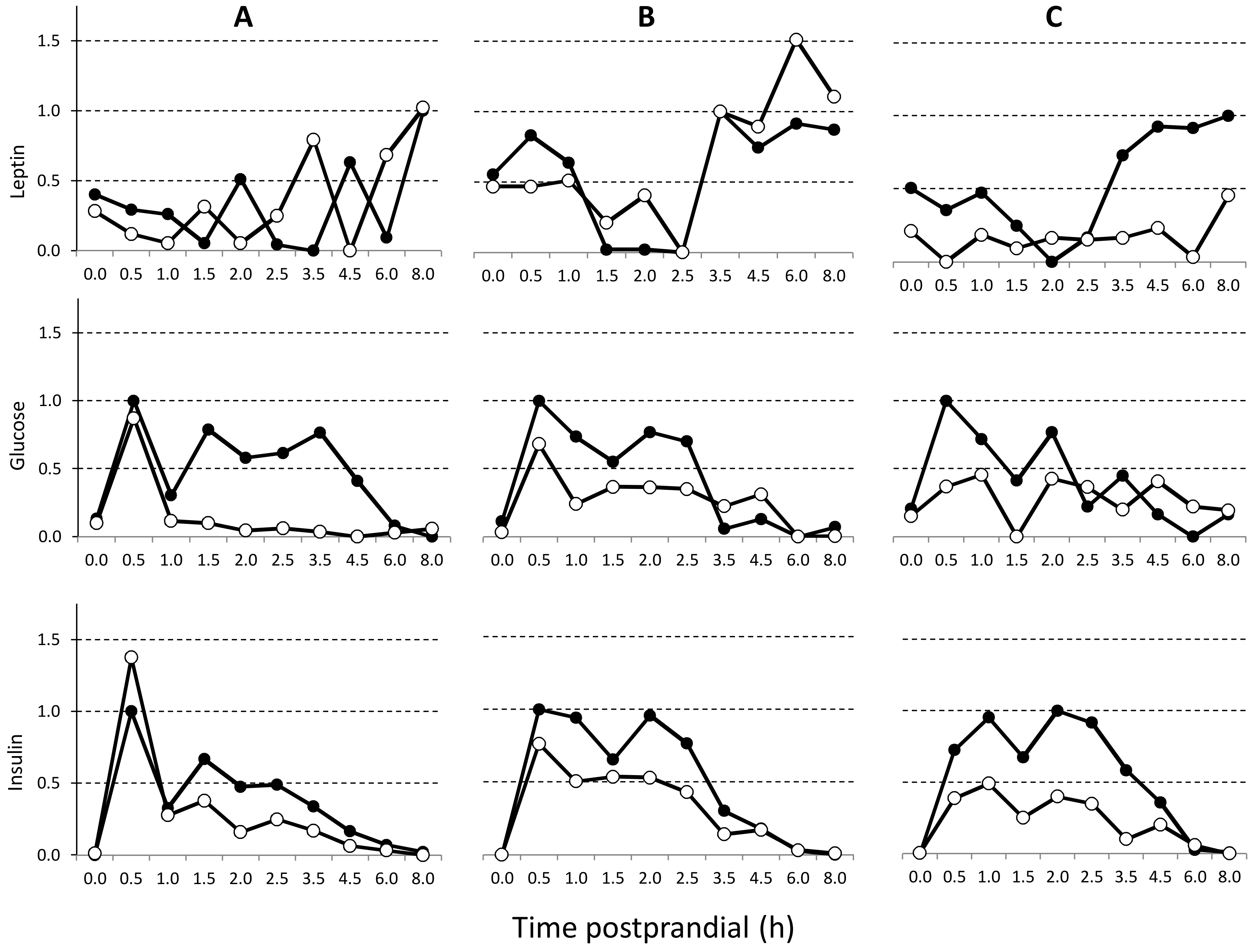

A
B
C
Leptin
Glucose
Insulin
1.5
1.0
0.5
0.0
0.0 0.5 1.0 1.5 2.0 2.5 3.5 4.5 6.0 8.0
Time postprandial (h)

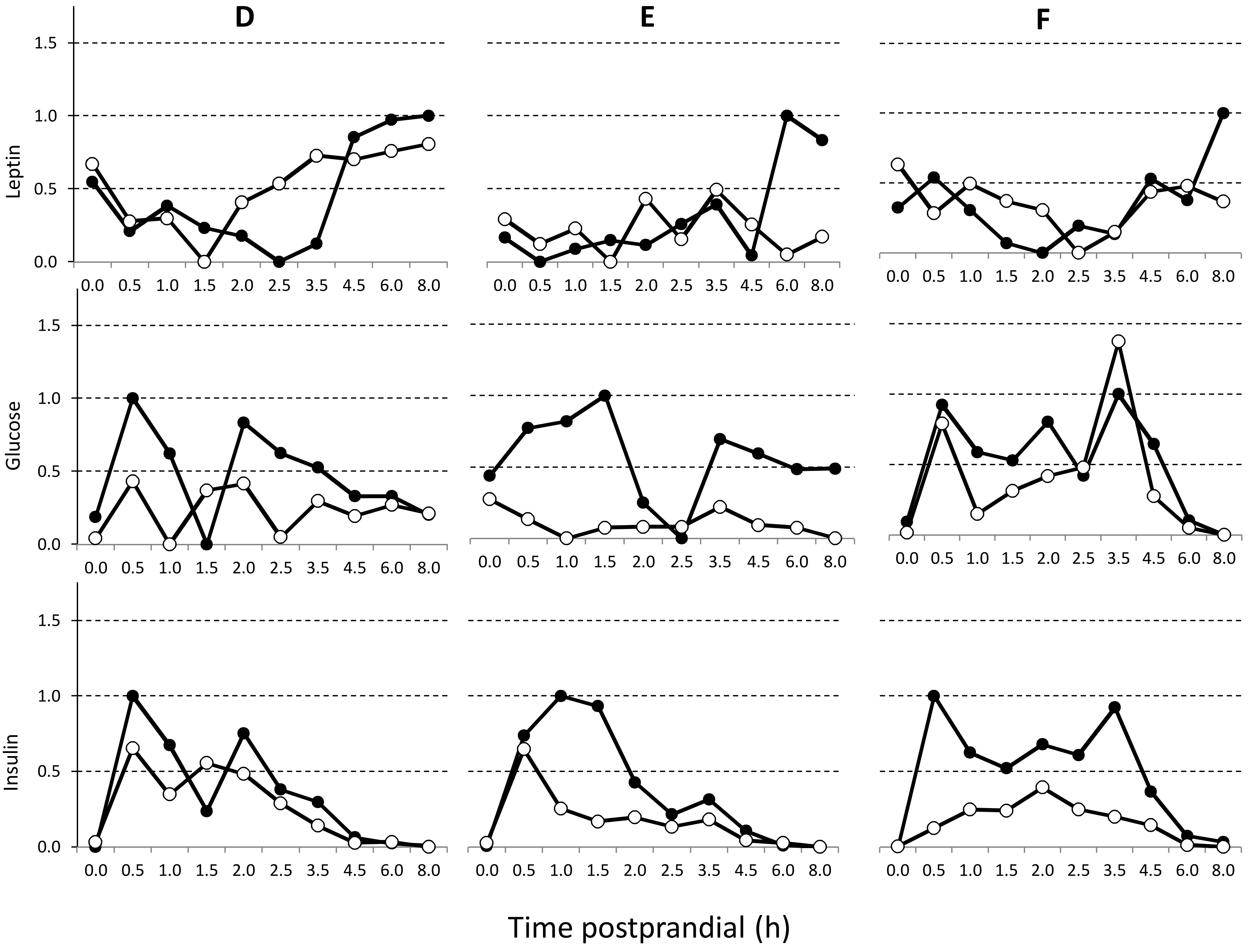
D
E
F
Leptin
Glucose
Insulin
0.0
0.5
1.0
1.5
2.0
2.5
3.5
4.5
6.0
8.0
Time postprandial (h)

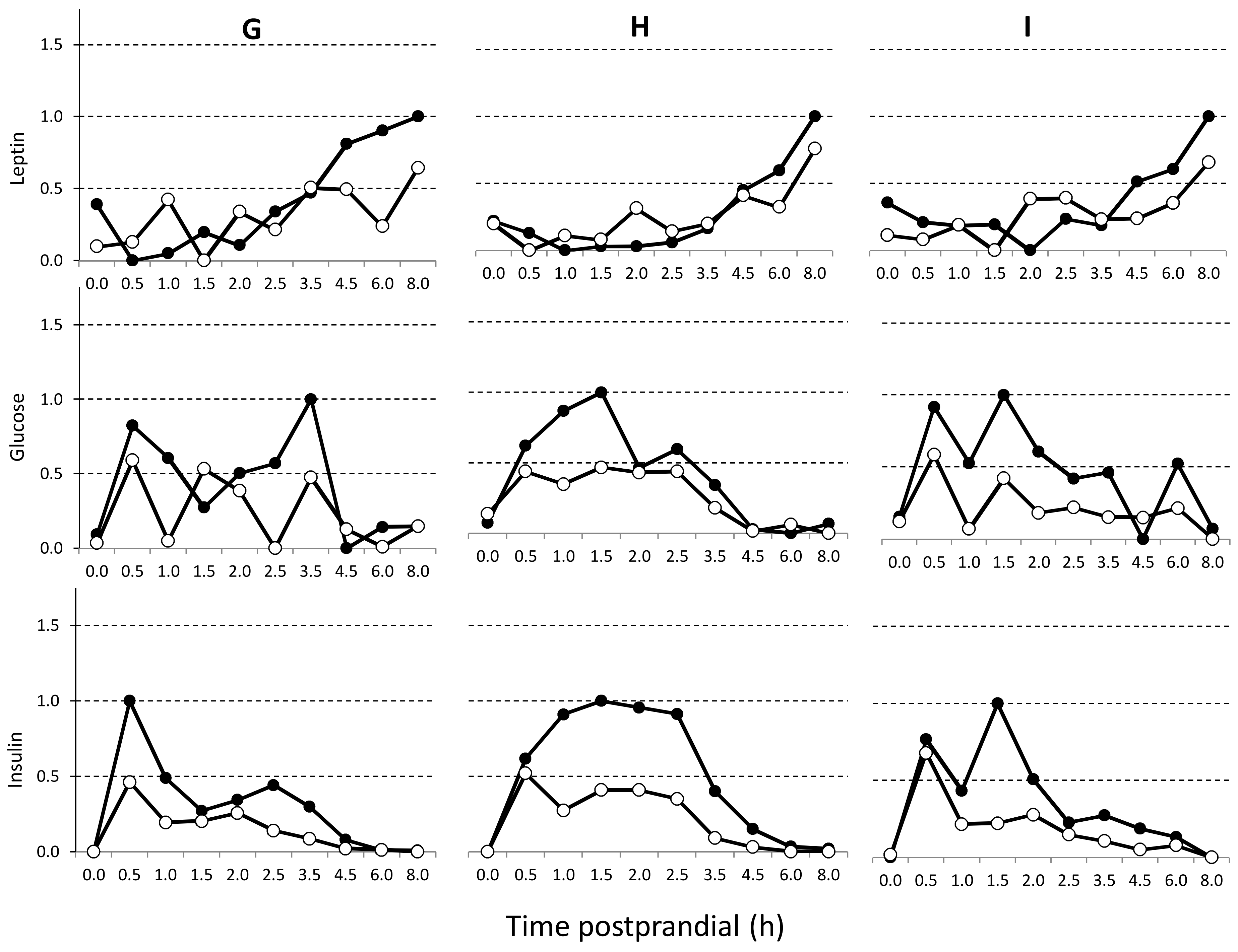

G
H
I
Leptin
Glucose
Insulin
1.5
1.0
0.5
0.0
0.0 0.5 1.0 1.5 2.0 2.5 3.5 4.5 6.0 8.0
Time postprandial (h)

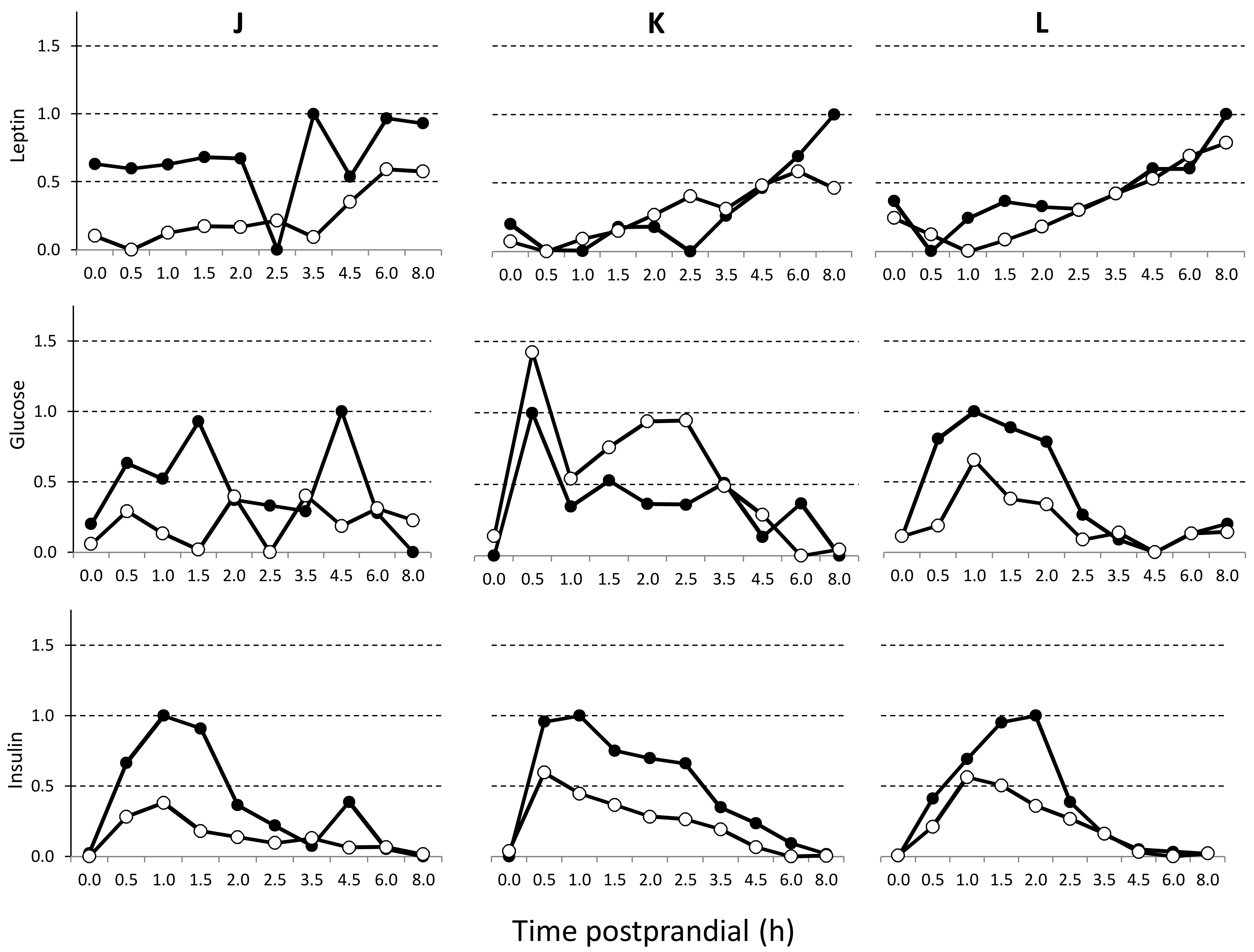

J
K
L
Leptin
Glucose
Insulin
0.0
0.5
1.0
1.5
0.0 0.5 1.0 1.5 2.0 2.5 3.5 4.5 6.0 8.0
Time postprandial (h)

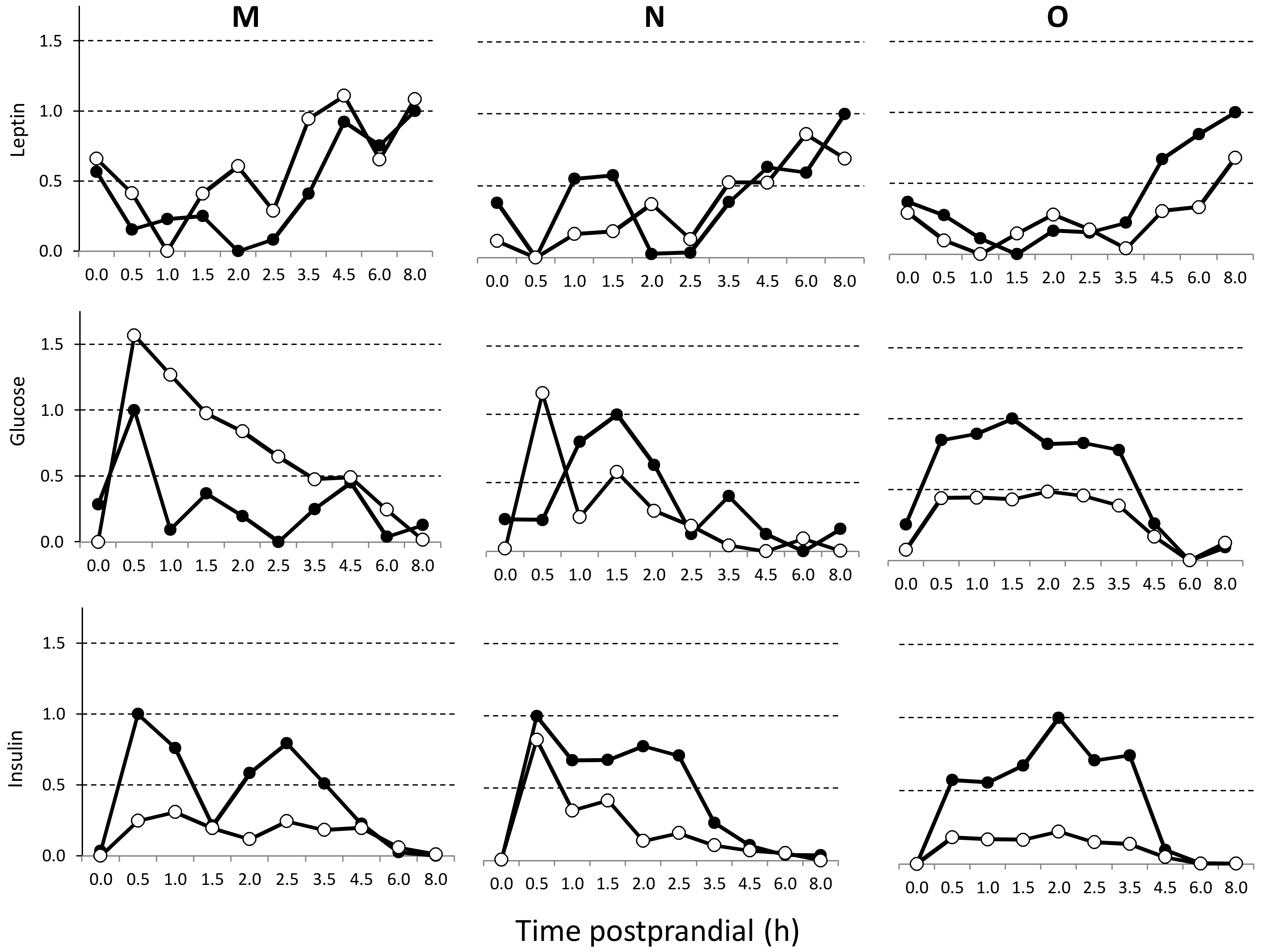
M
N
O
Leptin
Glucose
Insulin
0.0
0.5
1.0
1.5
2.0
2.5
3.5
4.5
6.0
8.0
Time postprandial (h)

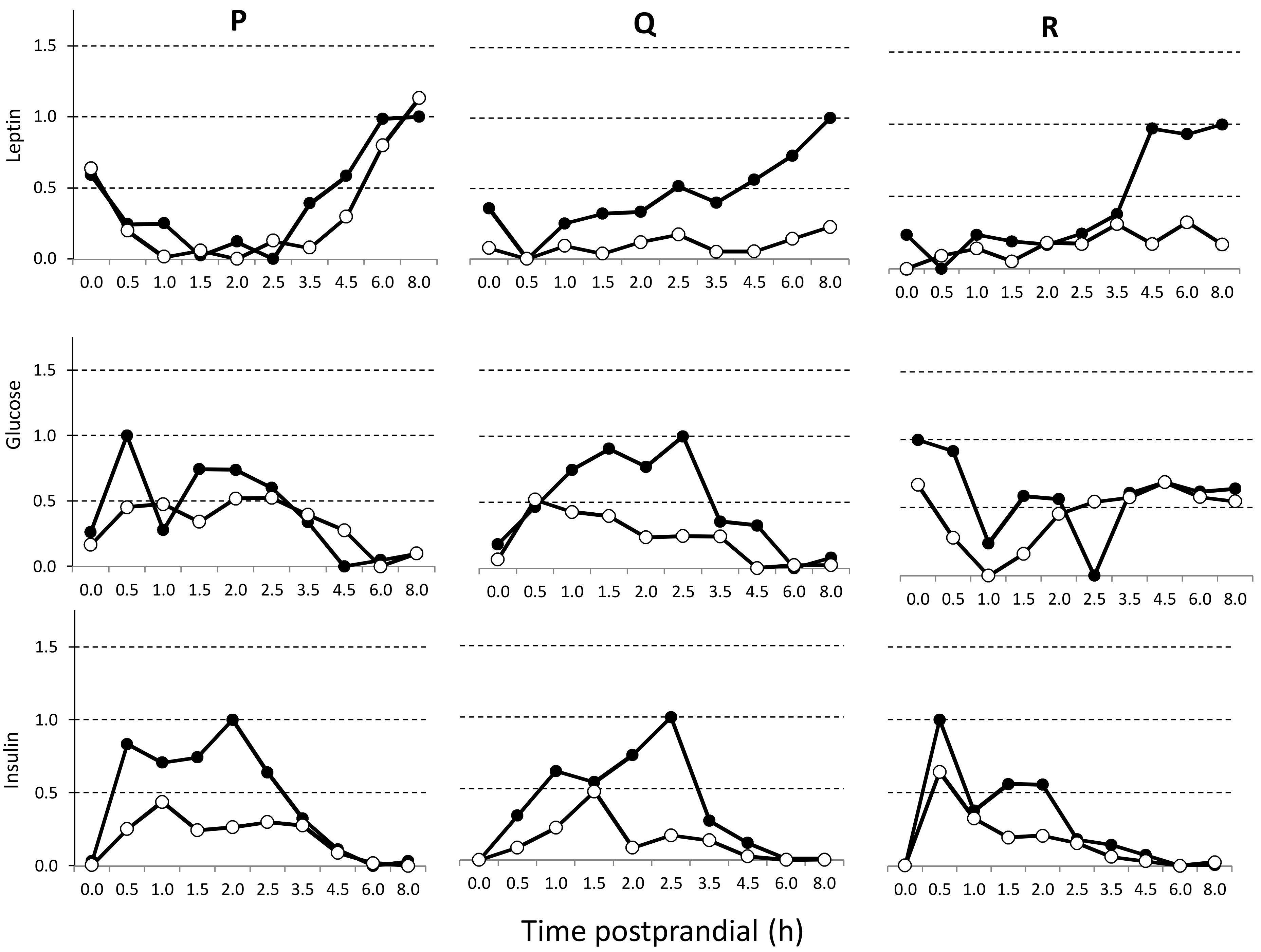

P
Q
R
Leptin
Glucose
Insulin
1.5
1.0
0.5
0.0
0.0 0.5 1.0 1.5 2.0 2.5 3.5 4.5 6.0 8.0
Time postprandial (h)

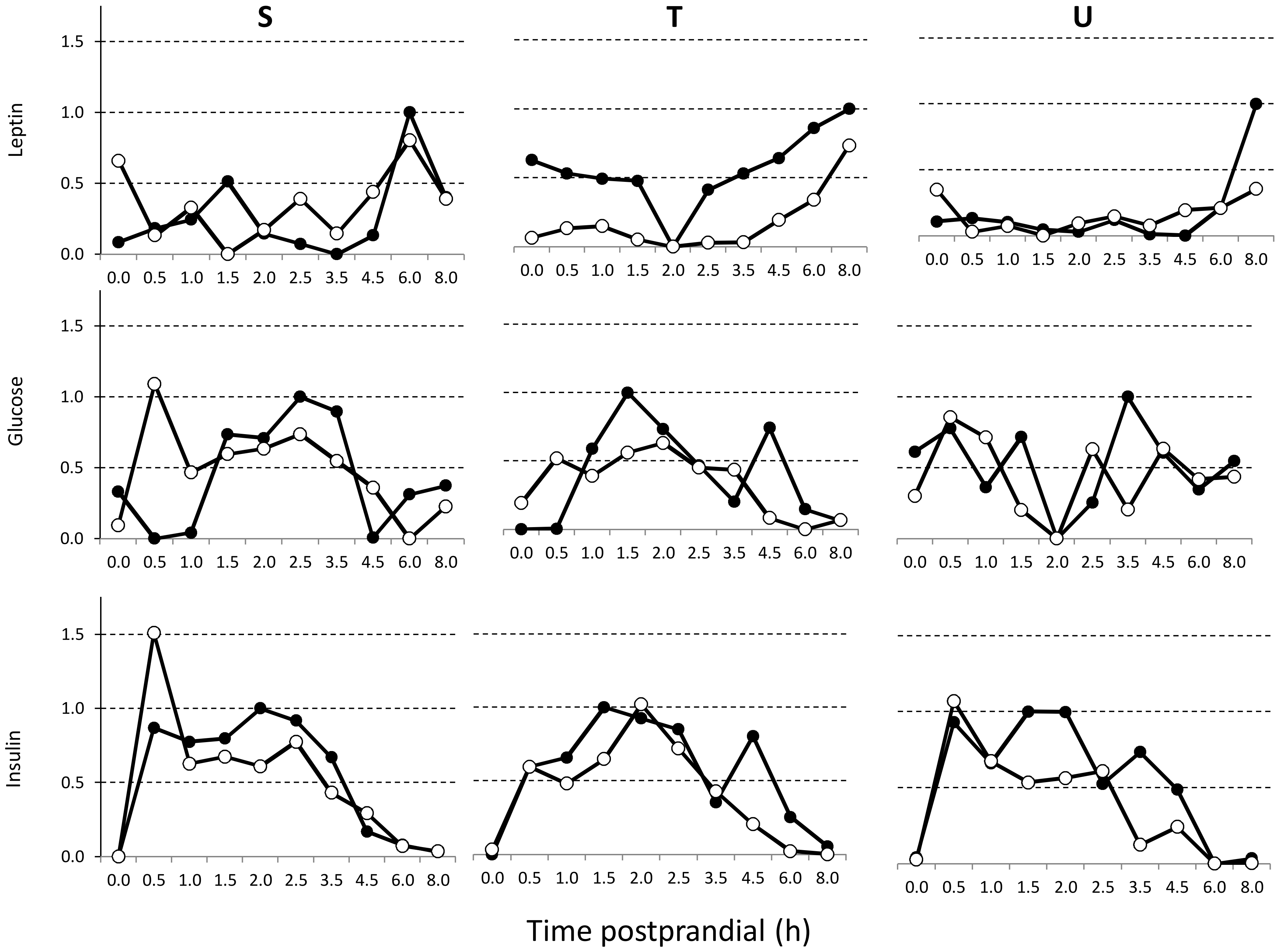
S
T
U
Leptin
Glucose
Insulin
0.0
0.5
1.0
1.5
0.0 0.5 1.0 1.5 2.0 2.5 3.5 4.5 6.0 8.0
Time postprandial (h)

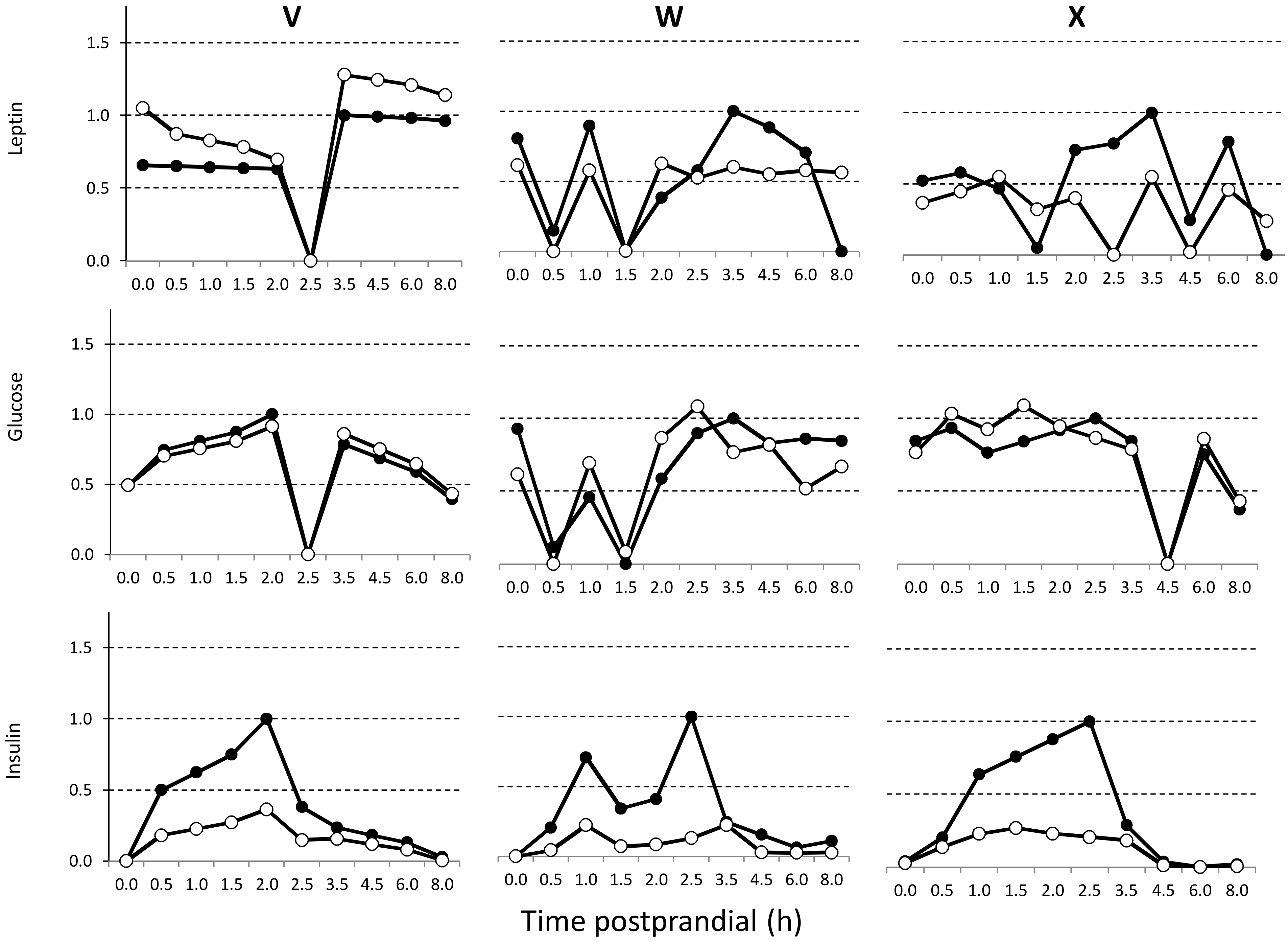
V
W
X
Leptin
Glucose
Insulin
0.0
0.5
1.0
1.5
0.0 0.5 1.0 1.5 2.0 2.5 3.5 4.5 6.0 8.0
Time postprandial (h)
