# Supplementary material for: Variation in metabolic responses to meal challenges differing in glycemic index in healthy women: Is it meaningful?
Source: Nutr Metab (Lond). 2012 Mar 29;9:26. doi: 10.1186/1743-7075-9-26 (PMC3352098; doi:10.1186/1743-7075-9-26)
Supplement: Additional file 4 — Appendix III: Mean Glucose AUC was lower under LGI conditions (p < 0.001), however the frequency distribution of the differential response to low and high glycemic meals were significantly different than expected (x2 = 6, n = 24, p = 0.014). The untransformed frequency distribution was best fit by a sum of 2 Gaussian equations. Using these constraints, 1 hypoglycemic, 16 normoglycemic, and 8 hyperglycemic relative responders were observed. These are highlighted in the inset of the lower figure, being unequally distributed about the mean regression of the HGI vs LGI response. [file 1743-7075-9-26-S4.PDF]

A

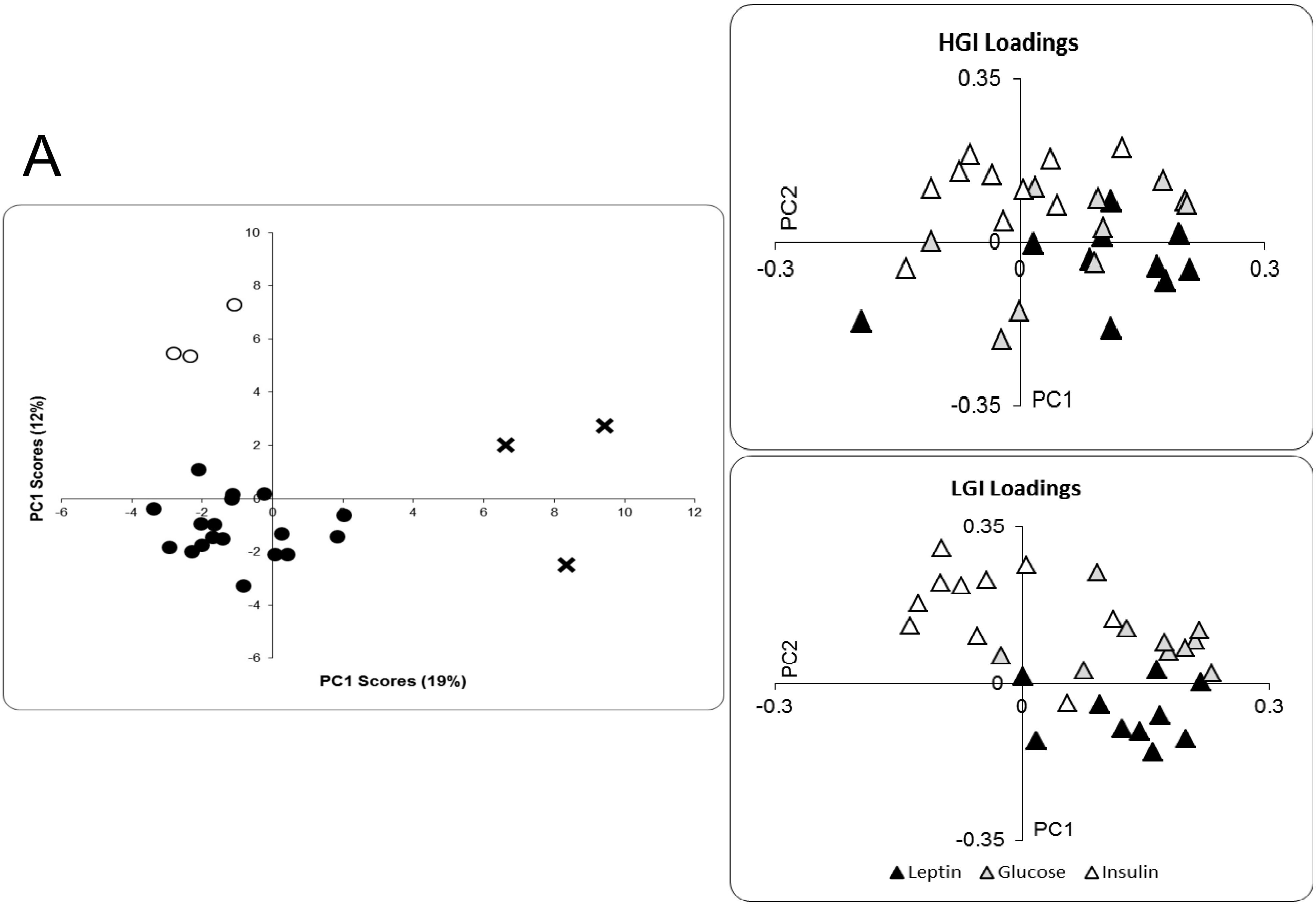

B

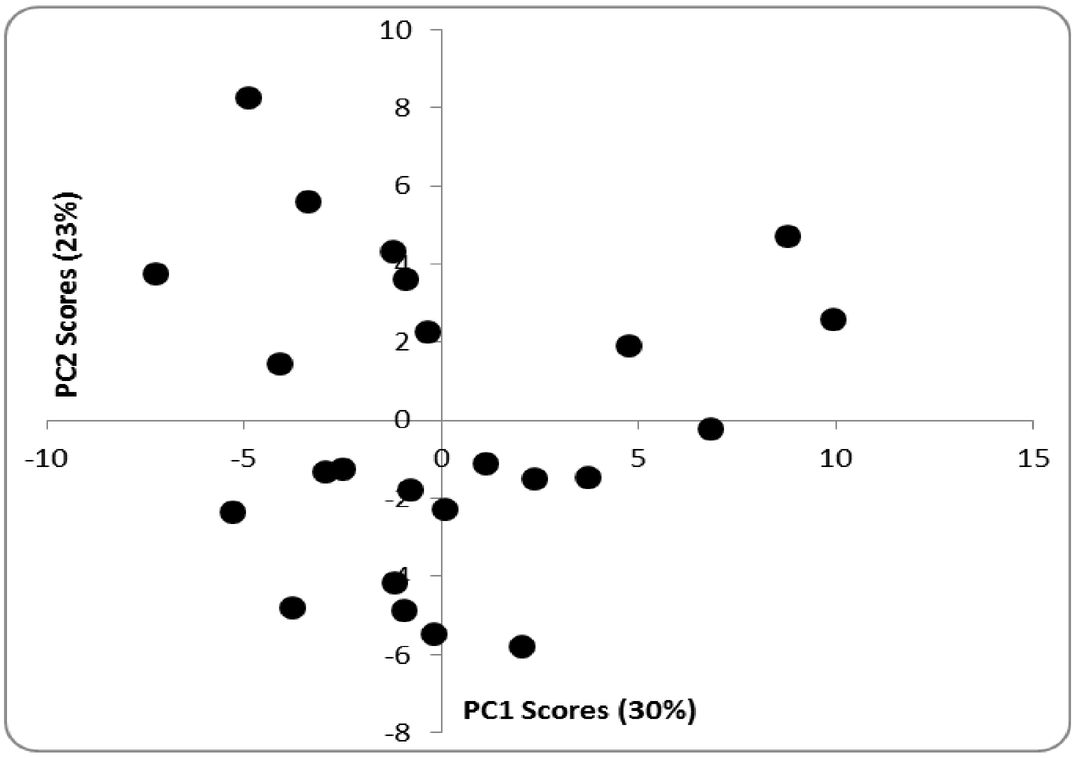

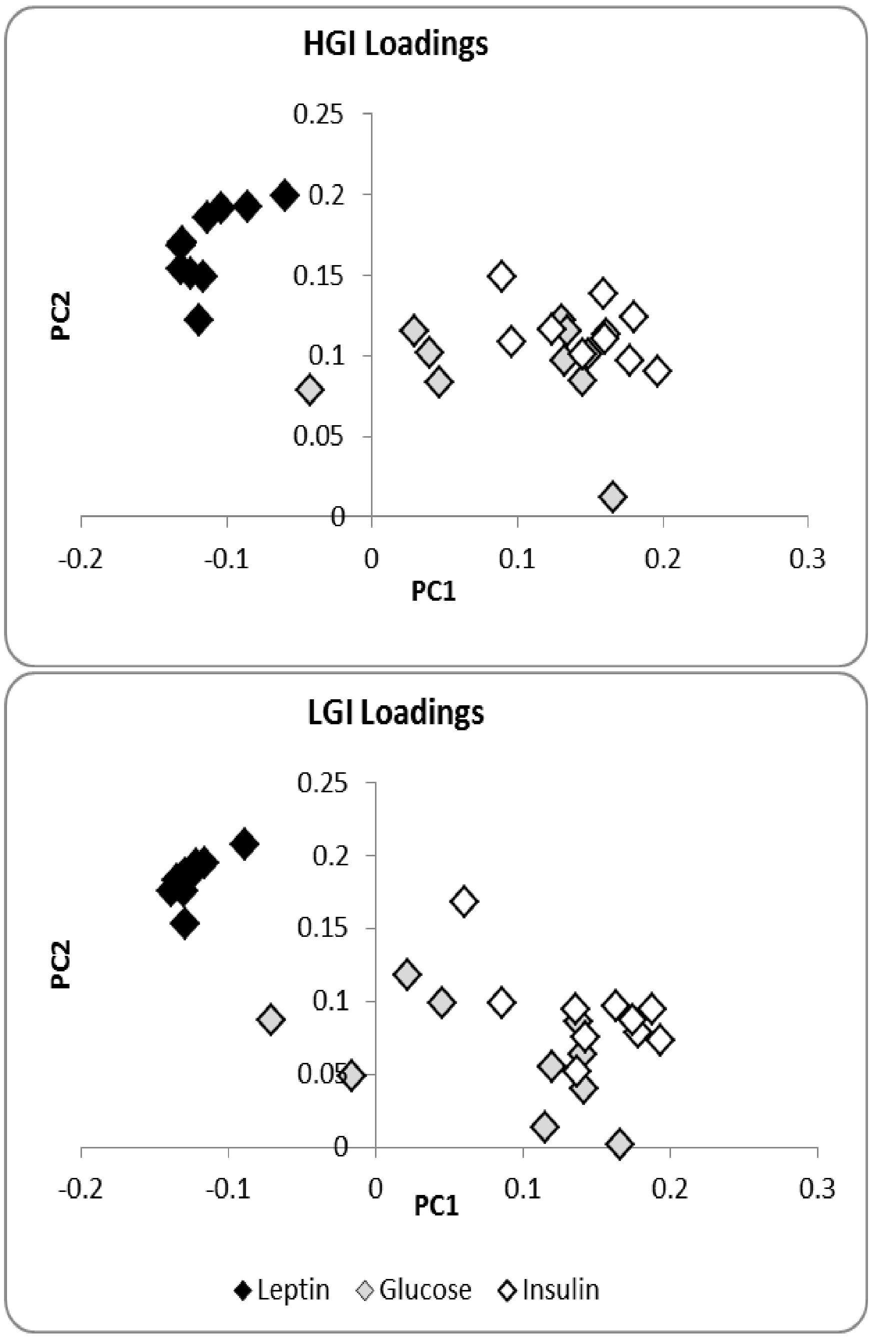

Panel A is a repeat of Fig 3 from the paper, and Panel B is the PCA scores and loadings plot of raw, unscaled/untransformed leptin, insulin and glucose data. In both high and low GI meal challenge responses, the distribution of the subjects in the scores plot appears to be dominated by the fact that the circulating concentration of leptin is very different from glucose and insulin. The scores plot using raw data in B does not afford a clear stratification of subjects, as opposed to panel A which uses nadir adjusted range scaled data where three clusters appear.
